# The Potato Yam Phyllosphere Ectosymbiont *Paraburkholderia* sp. Msb3 Is a Potent Growth Promotor in Tomato

**DOI:** 10.3389/fmicb.2020.00581

**Published:** 2020-04-21

**Authors:** Johannes B. Herpell, Florian Schindler, Mersad Bejtović, Lena Fragner, Bocar Diallo, Anke Bellaire, Susanne Kublik, Bärbel U. Foesel, Silvia Gschwendtner, Melina Kerou, Michael Schloter, Wolfram Weckwerth

**Affiliations:** ^1^Molecular Systems Biology (MOSYS), Department of Functional and Evolutionary Ecology, University of Vienna, Vienna, Austria; ^2^Vienna Metabolomics Center (VIME), University of Vienna, Vienna, Austria; ^3^Division of Structural and Functional Botany, Department of Botany and Biodiversity Research, University of Vienna, Vienna, Austria; ^4^Research Unit for Comparative Microbiome Analysis, German Research Center for Environmental Health, Helmholtz Zentrum München, Neuherberg, Germany; ^5^Archaea Biology and Ecogenomics Division, Department of Functional and Evolutionary Ecology, University of Vienna, Vienna, Austria

**Keywords:** *Paraburkholderia*, *Dioscorea*, plant-growth-promotion, phyllosphere, yams, acumen, plant-microbe-interaction, epiphyte

## Abstract

The genus *Paraburkholderia* includes a variety of species with promising features for sustainable biotechnological solutions in agriculture through increasing crop productivity. Here, we present a novel *Paraburkholderia* isolate, a permanent and predominant member of the *Dioscoreae bulbifera* (yam family, *Dioscoreaceae*) phyllosphere, making up to 25% of the microbial community on leaf acumens. The 8.5 Mbp genome of isolate Msb3 encodes an unprecedented combination of features mediating a beneficial plant-associated lifestyle, including biological nitrogen fixation (BNF), plant hormone regulation, detoxification of various xenobiotics, degradation of aromatic compounds and multiple protein secretion systems including both T3SS and T6SS. The isolate exhibits significant growth promotion when applied to agriculturally important plants such as tomato, by increasing the total dry biomass by up to 40%. The open question about the “beneficial” nature of this strain led us to investigate ecological and generic boundaries in *Burkholderia sensu lato*. In a refined phylogeny including 279 *Burkholderia sensu lato* isolates strain Msb3 clusters within Clade I *Paraburkholderia*, which also includes few opportunistic strains that can potentially act as pathogens, as revealed by our ecological meta-data analysis. In fact, we demonstrate that all genera originating from the “plant beneficial and environmental” (PBE) *Burkholderia* species cluster include opportunists. This indicates that further functional examinations are needed before safe application of these strains in sustainable agricultural settings can be assured.

## Introduction

Plants harbor diverse microbial communities whose members can establish a wide range of beneficial interactions with their host. At the root level many of these interactions have been heavily studied (Philippot et al., 2013) and the findings, usually concerning facultative mutualistic associations, have revolutionized our understanding of plants and agricultural processes. Much less focus has been directed toward the phyllosphere of plants, and despite findings outlining the importance of this habitat, our understanding of it is still lacking behind while the major focus is still on understanding plant-pathogen interactions (Vorholt, 2012).

Certain bacterial groups are commonly found in plant shaped environments, as for example members of the *Burkholderia sensu lato* (*s.l.*) species complex. Many members of *Burkholderia s.l.* have been isolated and observed in association to plants; some as pathogens (Burkholder, 1942, 1950; Lee et al., 2005) or as plant growth promoting rhizobacteria (PGPR) and β-rhizobia (Estrada-De Los Santos et al., 2001; Compant et al., 2008; Suarez-Moreno et al., 2012) and others as secondary metabolite producing wardens of their host plant (Van Oevelen, 2002; Lemaire et al., 2011; Carlier and Eberl, 2012; Lemaire et al., 2012a, b; Sieber et al., 2015; Carlier et al., 2016; Pinto-Carbo et al., 2018). The later are of exceptional nature, as they usually colonize glandular structures within the leaf mesophyll, referred to as “leaf-nodules.” These phyllosphere colonizers affiliated with the genus *Caballeronia* are thought to produce secondary metabolites to protect their hosts from herbivores. These obligate symbionts are uncultivable and have largely reduced genomes (Carlier and Eberl, 2012; Pinto-Carbo et al., 2016). This discovery gave great relevance to the leaf as an important habitat for beneficial interactions and to *Burkholderia s.l.* species as beneficial interaction partners. The specificity of the symbiosis, however, confines efforts trying to transfer these traits or species to economically important crop plants. Recently, the occurrence of the leaf nodule symbiosis has been described in the yam family, *Dioscoreaecea* (Carlier et al., 2017; Pinto-Carbo et al., 2018; De Meyer et al., 2019). The sansibar yam, *Dioscorea sansibarensis*, is considered the only species of the monocot family, in which nodule formation occurs, and interestingly, the facultative symbiont, *Orrella dioscoreae*, carries a large genome and can be cultured. It belongs to the family *Alcaligenaceae* within the order *Burkholderiales*. In this interaction the bacteria invade a pre-existing gland, formed by a folding of the upper-epidermis on the leaf acumen. Trichomes develop into the bacteria-filled lumen. They are thought to play a role in the exchange of nutrients (Miller and Reporter, 1987; Carlier et al., 2017).

In the present study we investigate the prevalence of a bacterial strain of the genus *Paraburkholderia* in the phyllosphere of the potato yam, *Dioscorea bulbifera* (L.), an edible yam species cultivated in large parts of the tropics due to its potato-like aerial tubers. Similar to *D. sansibarensis*, *D. bulbifera* possesses prominent leaf acumens and they are densely covered with secretory trichomes. As most other known cases of leaf symbiosis are conserved throughout entire lineages (Lemaire et al., 2011), we hypothesized that the tendency of *D. sansibarensis* to engage in beneficial associations with bacterial partners may be mirrored in closely related non-nodulated *Dioscorea* species. Indeed, we detected and isolated *Paraburkholderia* sp. strain Msb3 from leaf acumens of *Dioscorea bulbifera*. Here, we describe its ecology and its potential. To gain insights into its role on its host plant, its genetic and metabolic repertoire and its potential for agricultural applications we conducted targeted amplicon sequencing experiments on its host, sequenced and annotated its genome, determined its phylogenetic position and tested its performance in plant growth assays. We hypothesize that this association represents an early stage in the development of a more intimate symbiotic association as described in *D. sansibarensis* and propose to use strain Msb3 as a model to study the genetic prerequisites for such a development and to determine its effect on other plant hosts. Additionally, to tackle safety concerns associated with bacteria of the *Burkholderia s.l.* species complex, we analyze a large amount of metadata in order to map opportunistic pathogenicity within *Burkholderia s.l.* onto a robust phylogenetic framework and challenge many earlier generalizations.

## Materials and Methods

### Specimen Sampling

Several *D. bulbifera* individuals were raised from bulbils and were grown in the greenhouse under natural photoperiods at 25 ± 5°C. The parent bulbil has been propagated in the greenhouse through reproductive underground tubers for a number of years before the present study. The plants used for the present study were raised in 20 L pots containing seven parts garden soil (150−600 mg L^–1^ N, 150−600 mg L^–1^ P_2_O_5_, 200−900 mg L^–1^ K_2_O, 150 mg L^–1^ Mg, pH = 5–6.5) (*Franz Kranzinger GmbH, Spezialerden-Erzeugung, Straßwalchen, Austria*), one part perlite and one part quartz sand (grain size 0.5 mm) and were consistently fertilized throughout their growth period using 0.05% WUXAL^®^ Super (*Hauert MANNA Düngerwerke GmbH, Nürnberg, Germany*) consisting of 8% N (2.3% NO_3_^–^, 3.7% NH_4_^+^, 2% Urea), 8% P_2_O_5_, 6% K_2_O and trace elements in water (conductivity 0.51 mS). Leaves used for analysis were clipped from the vine. Experiments were conducted on greenhouse grown plants over a period of 3 years: isolation of bacteria and initial detection through amplification of target genes was performed during its growth period in autumn and winter of 2017/2018. Samples used for community profiling through amplicon sequencing and re-cultivation of strain Msb3 were taken in the subsequent natural growth period in the autumn of 2018. Samples used for microscopic analyses and localization of bacteria through general DNA staining procedures were harvested throughout both of these periods. Leaves used for fluorescence *in situ* hybridization (FISH) for the specific detection of *Burkholderia s.l.* species were harvested between December 2019 and February 2020.

### Isolation of Bacteria

Fresh leaves were rinsed briefly with water and subsequently divided into ca. 2 cm x 4 cm pieces. The pieces were surface-sterilized in a 2.8% (w/v) sodium hypochlorite (NaClO) solution for 5 min, in ethanol (EtOH) for 1 min and sterile ddH_2_O for 1 min. Leaf acumens and sections were carefully ground with sterile mortar and pestle in 2 mL of sterile 0.4% sodium chloride solution. Serial dilutions were plated on tryptic soy agar medium (TSA) (7.5 g agar in 1 L tryptic soy broth medium, TSB) (*Carl Roth GmbH & Co., KG, Karlsruhe, Germany*) and incubated aerobically at room temperature (RT) (ca. 22°C) in the dark for 48 h. Single bacterial colonies were transferred to a new TSA plate and clonal colonies were used for colony PCR. Bacteria were stored in 30% glycerol in TSB at −80°C after initial identification. All subsequent analyses were done with bacteria grown from a cryo-stock batch generated from a clonal colony directly after its identification to ensure plasmid preservation.

### Physico-Chemical Characterization of Strain Msb3

The effect of different temperatures and antibiotics was examined by growing bacteria on plate (TSA) as well as in liquid culture (TSB). Growth on TSA was used to determine temperature boundaries, then growth rates at different temperatures were determined through comparison of the optical density (OD) at 600 nm of bacteria that were inoculated to a starting OD_600_ of 0.05, grown in 200 mL TSB in Erlenmeyer flasks, steadily shaking at 200 rpm for 24 h. All conditions were grown in triplicates and technical triplicates were measured for individual flasks. Temperatures tested included 20°C, 22°C, 25°C, 30°C, 35°C, 36°C, 37°C, and 42°C. The same approach was used to determine the minimal inhibitory concentrations of following antibiotics: ampicillin (50−200 μg/mL); cefalexin (10−40 μg/mL); chloramphenicol (12.5−50 μg/mL); carbenicillin (50−100 μg/mL); streptomycin (25−50 μg/mL); and kanamycin (50−200 μg/mL).

Growth enabled by utilization of a specific substrate as sole carbon source was determined by comparison of the OD_600_ following growth in 1 × M9 salts minimal medium (*Sigma-Aldrich Handels GmbH, Vienna, Austria*) containing 2 mM MgSO_4_ and 0.1 mM CaCl_2_ with and without addition of test substrate, comparing relative growth after 16 h to glucose (100%) as a control.

### DNA Extraction From Pure Culture

Strain Msb3 was grown from cryo-stock in liquid culture overnight at RT in TSB (*Carl Roth GmbH & Co., KG*). 4 mL (OD_600_ = 0.8) were transferred into a 15 mL tube and centrifuged at 2400 × *g* for 8 min to sediment biomass. Cells were washed in TE and subsequently lysed using lysozyme (*Thermo Fisher Scientific, Waltham, MA, United States*). Lytic activity was stopped using Proteinase K (*Thermo Fisher Scientific*). This approach was chosen to prevent excessive shearing of DNA molecules in order to produce long sequencing reads. Extraction of total chromosomal and plasmid DNA was performed following a standard phenol/chloroform/isoamyl alcohol (PCI) extraction protocol as described below.

### DNA Extractions From Plants

Leaves and leaf acumens of *D. bulbifera* were either surface sterilized with NaClO for 3 min, followed by EtOH for 1 min and washed with ddH_2_O (9 × 30 s) or left untreated. Due to insufficiently clean DNA for reproducible downstream applications using standard procedures we comprised a new PCI (*Thermo Fisher Scientific*) based extraction protocol for clean nucleic acid extraction from plants. The crude plant extract is mixed with a polyvinylpolypyrrolidon (PVPP) suspension which binds tannins and polyphenols and the mixture can subsequently be removed with particles through centrifugation. The method presented here is a modified version of the PVPP based protocol presented by Kasajima et al. (2013) and relies on the same principal. DNA was extracted from 25−100 mg of dried or frozen leaf tissue. The homogenized tissue was mixed and washed with alkaline PVPP buffer (Tris–HCl, pH 9.5, 50 mM; EDTA, 10 mM; NaCl, 4 M; CTAB, 1%; PVPP, 0.5%; 2-mercaptoethanol, 1%) and the supernatant was used for further DNA extraction following a standard PCI protocol. Chloroform phase separation was repeated three times to enhance phenol residue removal. DNA was precipitated in 0.03 M NaOAc and 70% EtOH at −20°C or 0.05 M NaCl and isopropanol at RT for 1 h and stored in ddH_2_O at −20°C.

### PCR Amplification and Sequencing of *gyrB* and 16S rRNA Genes

DNA extracted from *D. bulbifera* as well as colonies grown on agar plate were used as a template for amplification. Primers 27F/1492R (Frank et al., 2008) were used to amplify 16S rRNA genes. For the amplification of *gyrB*-genes the genus specific primer pair “gyrB,” designed by Spilker et al. (2009) for the genus *Burkholderia s.l.* was used. All oligonucleotides used in this study were obtained from Microsynth (*Microsynth AG, Balgach, Switzerland*) and are summarized in Table 1.

**TABLE 1:** Oligonucleotides used in the present study.

**Target group**	**Name**	**Target sites**	**Sequence (5′–3′)**	**Modification (3′ and 5′)**	**Purpose**	**References**
Bacteria	EUB338-I	16S rRNA	GCTGCCTCCCGTAGGAGT	ATTO 488	FISH	Amann et al., 1990
*Planctomycetales*	EUB338-II	16S rRNA	GCAGCCACCCGTAGGTGT	ATTO 488	FISH	Daims et al., 1999
*Verrucomicrobia*	EUB338-III	16S rRNA	GCTGCCACCCGTAGGTGT	ATTO 488	FISH	Daims et al., 1999
Bacteria	NON-EUB	non-specific	ACTCCTACGGGAGGCAGC	ATTO 488	FISH	Wallner et al., 1993
*Burkholderia s.l.*	Burkho	16S rRNA	ACCCTCTGTTCCGACCAT	Cy3	FISH	Hogardt et al., 2000
*Burkholderia s.l.*	gyrB F	gyrB	ACCGGTCTGCAYCACCTCGT		PCR	Spilker et al., 2009
*Burkholderia s.l.*	gyrB R	gyrB	YTCGTTGWARCTGTCGTTCCACTGC		PCR	Spilker et al., 2009
Bacteria	335F	16 rDNA	CADACTCCTACGGGAGGC		PCR	Dorn-In et al., 2015
Bacteria	769R	16 rDNA	ATCCTGTTTGMTMCCCVCRC		PCR	Dorn-In et al., 2015
Bacteria	27F	16 rDNA	AGAGTTTGATCMTGGCTCAG		PCR	Frank et al., 2008
Bacteria	1492R	16 rDNA	TACGGYTACCTTGTTACGACTT		PCR	Frank et al., 2008

Amplification of target DNA with the DreamTaq DNA Polymerase (*Thermo Fisher Scientific*) was performed in a 30 μL reaction volume, consisting of 21.05 μL sterile ddH_2_O, 6 μL 10x Dream Taq Green Buffer (incl. 20 mM MgCl_2_), 0.6 μL dNTP Solution Mix (10 mM of each dNTP, *New England Biolabs, Ipswich, MA, United States*), 0.15 μL Dream Taq DNA Polymerase and 0.6 μL 10 mM forward and 0.6 μL 10 mM reverse primer as well as 1 μL template DNA (20 ng/μL for plant extracts and 1 ng/μL for pure culture controls) or a colony lysed in sterile ddH_2_O. The reaction conditions were as follows: one cycle at 94°C (3 min); 30 cycles at 94°C (30 s), 50°C/60°C (30 s) for 16S rRNA/gyrB, respectively, and 72°C (1 min/2 min for 16S rRNA/gyrB respectively), plus one final cycle at 72°C (6 min).

Amplification with the Phire Hot Start II Polymerase (*Thermo Fisher Scientific*) was performed in 20 μL reaction mix consisting of 11.6 μL sterile ddH_2_O, 4 μL 5 x Phire Green Reaction Buffer (1.5 mM MgCl_2_ final concentration), 0.4 μL dNTP Solution Mix (10 mM of each dNTP), 0.4 μL Phire Hot Start II Polymerase and 1 μL 10 mM forward and 1 μL 10 mM reverse primer as well as 1 μL template DNA (20 ng/μL for plant extracts and 1 ng/μL for controls) or a colony lysed in sterile ddH_2_O. The reaction conditions were as follows: one cycle at 98°C (2 min); 30 cycles at 98°C (10 s), 48°C/60°C (10 s) for 16S rRNA/gyrB, respectively, and 72°C (20 s/8 s for 16S rRNA/gyrB respectively). Resulting PCR products were purified using a Monarch^TM^ PCR/DNA clean up Kit (*New England Biolabs*) and Sanger sequenced by Eurofins Genomics (*Eurofins Genomics Germany GmbH, Ebersberg, Germany*).

### Amplicon Sequencing

Plants were grown in five biological replicates and three leaves per plant were pooled for further analysis. Leaves were divided into two equal parts and one half was surface sterilized, the other half was left untreated. The acumen was always removed and handled individually. Additionally, we collected water that was used to wash the leaf surface from the acumen. DNA quality and quantity were measured via Fragment Analyzer^TM^ (*Advanced Analytical Technologies, Inc., Ankeny, United States*). Subsequently, PCR was performed in triplicate using the Phusion Flash High-Fidelity PCR Master Mix (*Thermo Fisher Scientific*) and primer pair 335F/769R (Dorn-In et al., 2015) to avoid co-amplification of plant and chloroplast DNA in a total volume of 10 μL (10 ng DNA template, 12.5 μL PCR master mix, 10 pmol of each primer). PCR conditions were 10 s at 98°C; 30 cycles of 1 s at 98°C, 5 s at 59°C, 45 s at 72°C; 1 min 72°C. To exclude potential contamination by PCR, a no template sample using water instead of DNA was included in all following steps. PCR products were qualified using gel electrophoresis pooled and purified using Agencourt AMPure^®^ XP beads (*Beckman Coulter, Brea, CA, United States*). DNA quality and quantity were checked as described above. The subsequent indexing PCR was performed using the Nextera XT Index Kit v2 (*Illumina, Inc. San Diego, CA, United States*) and the Phusion Flash high-fidelity PCR master mix (*Thermo Fisher Scientific*) in a total volume of 25 μL (10 ng DNA template, 12.5 μL PCR master mix, 2.5 μL of each index) and the following PCR conditions: 30 s at 98°C; 8 cycles of 10 s at 98°C, 30 s at 55°C, 30 s at 72°C; 5 min 72°C. Indexing PCR products were purified and quantified as described above and pooled in an equimolar ratio of 4 nM. Sequencing was performed using an Illumina Miseq platform (*Illumina Inc.*) with Reagent Kit v3 (600 cycles).

Sequences were analyzed using the QIIME 2 software package release 2018.8.0 (Caporaso et al., 2010) with default parameters. FASTQ files were trimmed and merged with a minimum read length of 50 and minimum Phred score of 15 using AdapterRemoval (Schubert et al., 2016). The QIIME 2 plugin DADA2 (Callahan et al., 2016) was used for quality control with the following parameters: 10 bp were removed n-terminally, reads were truncated at position 290 (forward) and 220 (reverse), respectively, and expected error was adjusted to 3. The resulting unique amplicon sequence variants (ASVs) are biological sequences discriminated from errors, allowing the detection of single-nucleotide differences over the sequenced gene. The ASV method has demonstrated a better specificity and sensitivity than OTU based methods, which cluster sequencing reads according to a fixed dissimilarity threshold (Callahan et al., 2017). Taxonomic analysis was performed using the SILVA_132_QIIME release 99% as reference database and the classify-consensus-blast option of QIIME 2, setting –p-perc-identity and –p-min-consensus to 0.9 each. PCR negative control showed no ASVs contamination during sample processing. Data was analyzed and visualized using the phyloseq package in R (McMurdie and Holmes, 2013).

### Genome Sequencing and Assembly

Library preparation and genome sequencing was conducted by the next generation sequencing (NGS) facility of the Vienna Biocenter Core Facilities GmbH (*VBCF, Vienna, Austria*) using a PacBio Sequel instrument (*Pacific Biosciences of California, Inc., Menlo Park, CA, United States*). Subreads were assembled by application of the HGAP4 pipeline (Chin et al., 2013). The quality of the genome assembly was evaluated using QAST v4.4 (Mikheenko et al., 2018). Linear chromosomes and plasmids were circularized using circlator (Hunt et al., 2015).

### Genome Annotation and Analysis

Rapid, automated gene prediction was performed using the Prokka software package (Seemann, 2014). Clusters of orthologous groups of proteins (COGs) were assigned using the COGsoft software (Kristensen et al., 2010) running COGtriangles to assign COGs and COGnitor to assign COG function by comparison to the COG database. Macromolecular systems like bacterial protein secretion systems were identified using the MacSyFinder program (Abby et al., 2014) and the sets of models and profiles described by Abby et al. (2016). Additionally, the assembly was uploaded to the web-based MicroScope platform for microbial genome annotation and comparative genomics (Vallenet et al., 2006^1^), where the automatic annotation was manually curated. Pseudogenes were identified and the product description was then curated for all genes considered in detail in this study. All >70% identity matches to a protein with an experimentally verified function were designated with the same product description unless other evidence overruled this annotation. All other product descriptions, except (conserved) hypothetical proteins, were preceded by “putative.” For most coding sequences (CDS) a >90% identity match was found with *P. xenovorans* strain LB400 with a very well manually curated genome (Chain et al., 2006). Unless new evidence overruled this annotation, it was designated with the same putative function.

### Phylogenomic Analysis

Available genomes and phylogenetic marker sequences of *Burkholderia*, *Paraburkholderia* and *Caballeronia* strains were retrieved from GenBank and the Joint Genome Institute’s Integrated Microbial Genomes database (JGI-IMG) as well as the SILVA rRNA database project and the PubMLST db (Jolley et al., 2018). Multiple sequence alignments were created using Mafft (Katoh et al., 2002) and trimmed using BMGE (Criscuolo and Gribaldo, 2010). Trimmed alignments were used to calculate maximum likelihood phylogenomic trees in IQ-Tree (Felsenstein, 1981) displaying ultrafast bootstrap (UF-BS) (Minh et al., 2013) and SH-aLRT (Guindon et al., 2010) values. Trees were manually annotated.

### Calculation of ANI Values

ANI values were determined using the JSpeciesWS web server (Richter et al., 2016). The assembly of the Msb3 genome was uploaded to the JSpeciesWS website in FASTA format together with whole genome sequences selected based on tetra-nucleotide usage. ANI analysis was performed with the ANIb algorithm, accessible via the JSpeciesWS web service.^2^

### Plant Growth Promotion Assays

Seeds of tomato (*Solanum lycopersicon* cv. Moneymaker) were surface sterilized by immersion in 70% (v/v) ethanol for 5 min followed by incubation in 2.8% NaClO for 10 min and subsequently washed with sterile distilled water for 30 min. The treated seeds were placed onto Murashige & Skoog (MS) agar medium including B5 vitamins (*Duchefa Biochemie B.V, Haarlem, Netherlands*) and germinated in the dark at 22°C. After 10 days seedlings were exposed to light and after 14 days uniform seedlings were selected and inoculated with bacteria. The seedlings were dipped into bacterial suspensions (0.5−5 × 10^9^ CFUs mL^–1^ in 1 × PBS either live or heat killed via autoclaving) and subsequently transferred to a 300 mL pot containing 60 g of sterilized garden soil (150−600 mg L^–1^ N, 150−600 mg L^–1^ P_2_O_5_, 200−900 mg L^–1^ K_2_O, 150 mg L^–1^ Mg, pH = 5–6.5) (*Franz Kranzinger GmbH*). The experiment was performed using nine biological replicates. In a second experiment using only three biological replicates the seedlings were first transferred to pots and after 7 days the soil was inoculated with bacteria. All pots were randomly placed in a growth chamber for 34 days at 22°C, a photoperiod of 16 h (100−130 PPFD, μmol m^–2^ s^–1^ at the ground level), and relative humidity of 65%. After 25 days all pots were fertilized once with 20 mL 0.1% WUXAL^®^ Super (*Hauert Manna Düngerwerke GmbH*) consisting of 8% N (2.3% NO_3_^–^, 3.7% NH_4_^+^, 2% Urea), 8% P_2_O_5_, 6% K_2_O and trace elements in distilled water (conductivity 0.51 mS). The plants inoculated with autoclave killed bacteria functioned as controls and were grown under the same conditions. The plants were harvested after 34 days. After removal of soil, total biomass fresh weight (FW) and dry weight (DW) as well as partial contributions of shoot and root were determined. Shoot length was measured as distance from its base to the last node of the emergence. Bacteria were re-cultivated from plant roots after homogenization of the tissue in 0.4% NaCl as described above.

### Microscopic Analyses

For scanning electron microscopic (SEM) analyses cell cultures were first dehydrated with EtOH (in a PBS EtOH mixture starting from 50% stepwise increasing by 10% to a final concentration of 100% EtOH) and subsequently CO_2_ critical point dried by application of a Leica EM CPD300 (*Leica Microsystems GmbH, Wetzlar, Germany*) automated critical point dryer. The dried samples were mounted on aluminum stubs and coated with gold for 80 s using a sputter coater (SCD 050; Denton Vacuum LLC). Finally, images were obtained in a JSM-6390 JEOL scanning electron microscope (*JEOL Germany GmbH, Freising, Germany*).

For production of fluorescent micrographs plant samples were fixed in 2% paraformaldehyde in 1 × PBS (pH = 7.2) for 1 h and subsequently washed in PBS, twice. Samples were then treated according to the respective downstream application. To keep as many bacterial cells on the leaf surface as possible we conducted a “minimal-washing” DNA staining experiment: Nucleic acids were stained by immersion of the specimen in 10 x SYBR^®^ < /*cps*:*sup* > -Green I (*Thermo Fisher Scientific*) in 1 × PBS for 10 min. Excess dye was removed through two additional washing steps. Samples could be imaged directly.

For specific identification of bacteria, FISH analysis was conducted as follows: Treatment with lysozyme solution (1 mg mL^–1^ in PBS) was applied for 10 min at 37°C and subsequently the samples were dehydrated in a stepwise ethanol series (25, 50, 75, and 99.9%; 15 min each step). Double labeling of oligonucleotide probes for FISH (DOPE-FISH) was performed with probes obtained from Microsynth (*Microsynth AG, Balgach, Switzerland*) labeled at both the 5′ and 3′ end positions with the respective fluorophore. An EUBmix targeting all bacteria consisting of an equimolar mixture of EUB338-I (Amann et al., 1990), EUB338-II, EUB338-III (Daims et al., 1999), coupled with the fluorochrome ATTO-488, and the *Burkholderia s.l*. specific probe Burkho (Hogardt et al., 2000), coupled with Cy3, were used for detection of the corresponding bacteria. The non-specific probe NON-EUB (Wallner et al., 1993) was used as a control for unspecific binding on separate samples. Hybridization was carried out at 46°C for 2 h or overnight within Eppendorf tubes containing the hybridization solution (20 mM Tris–HCl pH 8.0, 0.01% w/v SDS, 0.9 M NaCl, 10 ng μL^–1^ of each probe and 20% or 30% formamide for EUBmix and Burkho, respectively) and the respective plant sample. Post-hybridization was conducted at 48°C for 30 min with a pre-warmed post-FISH solution containing 20 mM Tris–HCl pH 8.0, 0.01% SDS, 5 mM EDTA pH 8.0 and NaCl at a concentration corresponding to the formamide concentration (0.215 M and 0.102 M NaCl for EUBmix and Burkho, respectively). When applied together, the probes were hybridized sequentially, starting with the probe requiring the higher formamide concentration. Samples were rinsed with ice cold distilled water before air drying in the dark. The samples were then observed under a confocal microscope.

Images were taken on a confocal laser scanning microscope (CLSM) (Leica DMRE SP5) (*Leica Microsystems*). A white light laser was used for excitation of fluorescence signals (SYBR^®^ < /*cps*:*sup* > -Safe: ex/em 410/520-540 nm; “chlorophyll autofluorescence”: ex/em 550 or 555/674-716 nm; “trichome specific signal”: ex/em 550 or 555/571-655 nm; Atto-488: ex/em 502/520-540 nm; Cy3: ex/em 550/560-590 nm). The scanning speed was 100 Hz and a 20× or 63× glycerol immersion objective was used for all images. Images were taken sequentially to prevent leaching of fluorescent signals into other channels. Stacks of 15 to 25 optical sections per series were recorded to cover a large range and the fluorescence data was combined in maximum projections using the Leica Application Suite (LAS) X (*Leica Microsystems*) software platform. Postprocessing included separation of dye signals from Cy3 and plant specific signals using the implemented “Channel Dye Separation” tool via signal informed selection of regions of interest. This was done due to the overlapping emission spectra of Cy3 and plant structures (compare Cy3 and trichome specific emission at excitation with 550 nm). Cy3-channel images and chlorophyll autofluorescence images were used as an input and the processed Cy3-channel image was used to generate montages shown in this manuscript. Non-processed images corresponding to the ones shown in the manuscript can be found in Supplementary Figure 3. Adaptation of brightness and contrast of all images to better suit print and screen representation was conducted through ImageJ version 1.52q (Schneider et al., 2012).

### Statistical Analysis

We tested for the significance of an inoculation effect on plant biomass and shoot length in the PGP assay in tomato as well as for a sample condition effect on bacterial read counts in the normalized amplicon sequencing dataset by applying Student’s *t*-tests. Reported *P*-values are calculated through two-tailed tests that assume a normal distribution of the data points and equal variance. To test for significant differences between means of abundance values in the normalized amplicon sequencing dataset we applied Tukey’s HSD (honestly significant difference) tests, based on the same assumptions. Values generated through analysis of independent biological replicates functioned as single data points used for the calculation. All analyses were performed in the program R (R Core Team, 2019), version 3.5.3.

Principal coordinate analyses were performed using pairwise, normalized, weighted UniFrac distances between all samples on the normalized ASV tables and the first two principal coordinates were visualized. Data was visualized through the R software packages phyloseq (McMurdie and Holmes, 2013) and vegan (Oksanen et al., 2013) using the plugin ggplot2 (Wickham, 2016).

## Results

### Isolation of Strain Msb3 From *D. bulbifera* Leaves

We realized the untapped potential of the leaf as a habitat for beneficial microbial interaction partners and conducted a broad screening in several plant families, including *Dioscoreaceae*, to detect novel forms of interactions in their phyllosphere. We investigated several *Dioscorea* species through amplification of marker genes using specific primers (Spilker et al., 2009). Sequencing of the PCR products revealed the presence of a highly abundant *gyrB* gene that clustered with that of free living cultivable *Paraburkholderia* species in DNA extracts of greenhouse grown *D. bulbifera*. In an effort to isolate the strain, serial dilutions of NaClO and EtOH surface sterilized leaves and leaf acumens were plated onto tryptic soy agar (TSA) medium and incubated at room temperature for 48 h. We obtained near to pure cultures from every leaf acumen extract in three biological replicates within two sequential experiments. No colonies were obtained on plates incubated with leaf extract, although the bacteria had been detected in the respective DNA extract (Supplementary Figure 1), likely a result of the harsh sterilization treatment. Amplification of 16S rRNA and *gyrB*-genes of individualized colonies and alignment to sequences amplified previously confirmed the identity of the isolate. The *Paraburkholderia* strain was designated Msb3.

We could reproduce cultivation of the strain from leaves harvested a second time in 2017 as well as after completion of an entire growth cycle of the perennial vine in 2018 and qualitative amplification of marker genes during that period showed consistent colonization (data not shown). The following results were obtained from mature leaves harvested in 2018.

### Strain Msb3 Dominates the *D. bulbifera* Acumen Surface Microbiome

To obtain a more detailed view of the microbial community in this environment and the role of strain Msb3 we conducted a 16S rRNA gene-based amplicon sequencing approach with general bacterial primers. The following samples were analyzed: leaves (L), surface-sterile leaves (LS), acumens (A), surface-sterile acumens (AS), and surface microbiota (−S). After analysis of raw reads, trimming, filtering and chimera removal (Supplementary Information), we defined a dataset consisting of 1,112 specific amplicon sequence variants (ASVs). ASVs occurring in extraction and PCR control samples as well as unassigned reads and singletons were removed and subsampling with 17,702 reads was performed, reflecting the lowest read number obtained per sample, to relatively compare chloroplast contaminations in different sample types. We defined a core dataset of 993 ASVs distributed across all samples through removal of plant-sequence-derived ASVs as well as ASVs present in less than 5% of all samples and normalized the final dataset by rarefying to 739 reads per sample. Rarefaction analysis indicated diversity coverage of >99% (Supplementary Figure 2A) and consequently an adequate sampling depth for further analysis.

**FIGURE 1:**
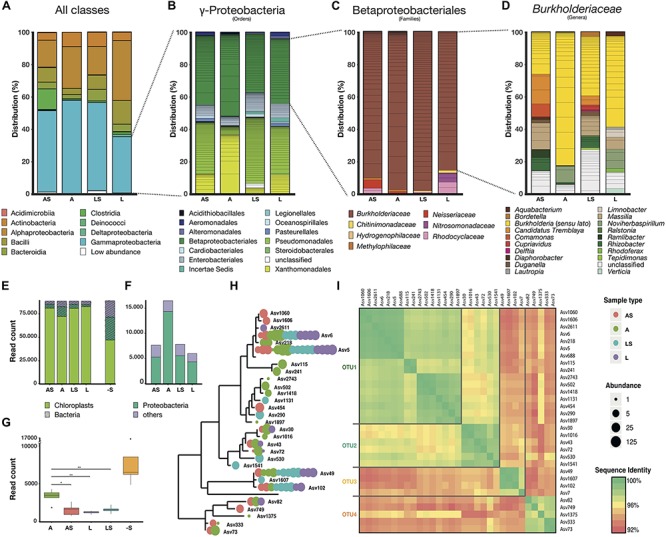
ASVs defining the leaf acumen microbiome. **(A)** Histograms showing the distribution of classes present in the 993 ASVs in samples from the *D. bulbifera* leaf lamina, untreated (L) and surface sterilized (LS), compared with classes present in those of the leaf acumen, (A and AS). The conditions were analyzed in five biological replicates and, per replicate, three leaves were pooled. **(B)** Distribution of orders present among the ASVs from the class γ-*Proteobacteria*. **(C)** Distribution of families present among the order *Betaproteobacteriales*, and **(D)** distribution of genera present in the family *Burkholderiaceae*. Taxonomic classification follows the current phylogenetic framework used by the SILVA database. **(E)** Bacterial load per sample type approximated through read count of bacterial ASVs compared to those of plant derived ASVs. **(F)** Histogram of total bacterial read count summed up per sample type and, **(G)** read count per sample displayed in a box-plot after removal of plant derived ASVs. Significant differences were determined through applying a two-tailed Student’s *t*-test, that assumes a normal distribution of the data points and equal variance, to read counts per condition in the normalized dataset. **(H)** Phylogenetic tree of ASVs that belong to the genus *Burkholderia* (*s.l*.). Their prevalence and abundance in certain sample types is represented by colored circles on the branch tip as shown in the legend on the right. **(I)** Sequence identity matrix of ASVs shown in panel **(H)** and the resulting OTU classification according to a 97% sequence identity threshold.

We explored the data in respect to the relative abundance of Msb3 and closely related taxa in different sample types. We compared all samples via principal coordinate analysis (Supplementary Figure 2B) to dissect major differences between location (L vs. A) and sample type (L vs. LS and A vs. AS, respectively). Surface microbiota, largely represented by γ-Proteobacteria (Supplementary Figure 2C), heavily influence community composition in A compared to AS. These differences become apparent in γ-Proteobacteria fractions (Figures 1A,B). It should be mentioned that we follow the taxonomy of the SILVA SSU Ref NR 99 release 132 in which β-Proteobacteria were reclassified as an order within γ-Proteobacteria following the Genome Taxonomy Database^3^ (Parks et al., 2018) and so, on an order level A is clearly different from other sample types: the *Xanthomonadales* subset is larger in A than in others (A vs. L: *p* = 0.0362; A vs. LS: *p* = 0.0111), but at the same time these are represented by fewer ASVs and *Pseudomonadales*, rather dominant in LS and AS, largely disappear (A vs. LS: *p* = 0.0015; A vs. AS: *p* = 0.0897). Generally, β-*Proteobacteriales* are almost exclusively made up of *Burkholderiaceae* (Figure 1C), whose distribution on a genus level (Figure 1D) is another driver for the separation of these samples. While disinfected samples, AS and LS, display a diverse set of evenly distributed genera within the family, both A and L show a less diverse pattern and especially A a higher fraction of *Burkholderia s.l*. (A vs. LS: *p* = 0.0498; A vs. AS: *p* = 0.0272). Here, *Burkholderia s.l.* make up 82% of *Burkholderiaceae* on average. In total *Burkholderia s.l.* species make up more than 25% of bacterial reads in A, constituting a substantial part. This becomes even more significant considering the bacterial load, approximated by relative abundance of bacterial reads compared to plant/chloroplast derived ASVs (Figure 1E). The summed number of reads per sample type is about two times higher than in other leaf derived samples (Figure 1F) and the read number per sample is consistently and significantly higher (A vs. L: *p* = 0.0052; A vs. LS: *p* = 0.0019; A vs. AS: *p* = 0.0345) (Figure 1G).

We also investigated the diversity and occurrence of ASVs classified as *Burkholderia s.l.* (Figure 1H) to identify Msb3 as well as other *Burkholderia s.l*. species. In total 31 ASVs were classified within the genus, and formed four distinct clusters (Figure 1I) in a sequence identity matrix as well as in a phylogenetic tree (Figure 1H). These were defined as operational taxonomic units (OTUs) based on a 97% sequence identity threshold for simplification. All sequences from OTU1 and OTU2 were identified as Clade I *Paraburkholderia* based on BLAST hits against NCBIs non-redundant nucleotide collection and compared to our own phylogenetic reconstructions (see section “Ecological and Phylogenetic Boundaries in *Burkholderia s.l.*”). OTU3 could not be identified unambiguously based on the 345 bp sequences but seems to be most closely related to some *Caballeronia* species and OTU4 is affiliated with Clade II *Paraburkholderia*. The most abundant ASVs (5, 6, and 218), which belong to OTU1, are at least 99.8% identical to the 16S rRNA gene of strain Msb3. In general, ASVs that belong to OTU1 are most abundant in A (but prevalent in all sample types) and are being reduced or disappear completely with surface sterilization, indicating that they are most abundant on the acumen surface.

### *Burkholderia s.l.* Species Aggregate on Secretory Trichomes

**FIGURE 2:**
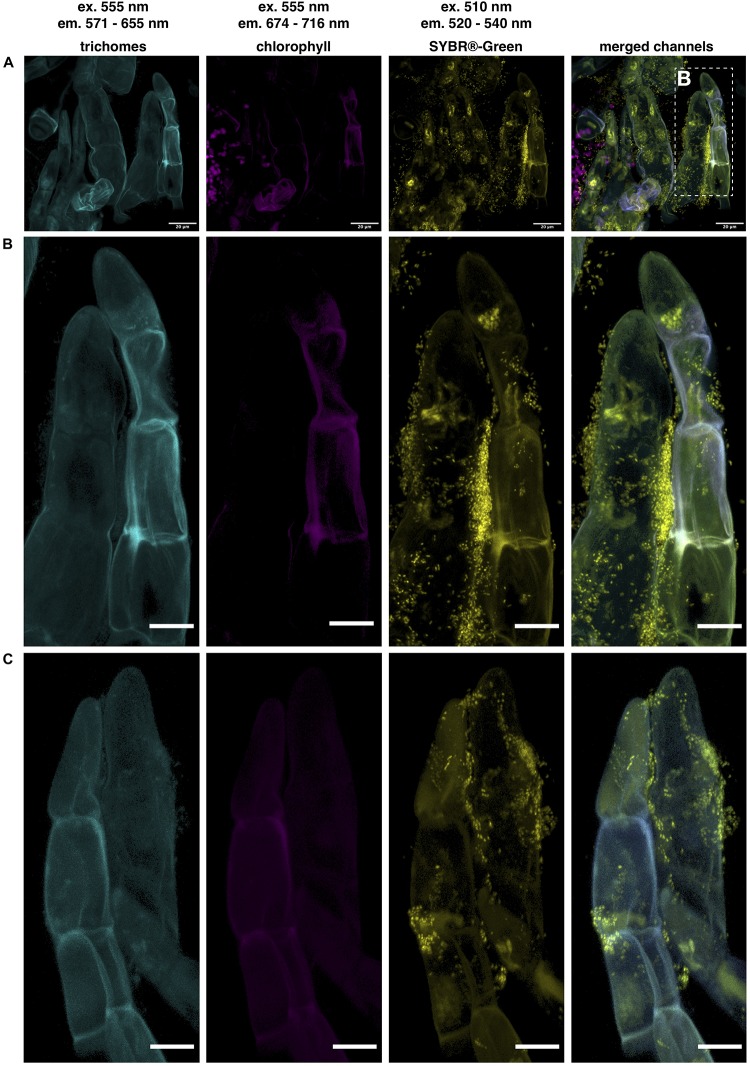
Microscopic analysis of bacteria associated to secretory trichomes on the *D. bulbifera* leaf acumen. Confocal laser scanning microscopy (CLSM) images of trichomes located on the upper side of the leaf acumen. Trichome autofluorescence could be separated from other channels and can be observed in cyan, chlorophyll autofluorescence in magenta and nucleic acids were stained with SYBR^®^ < /*cps*:*sup* > -Green, here depicted in yellow. Excitation (ex.) and emission (em.) wavelengths are indicated above. **(A)** Trichomes densely cover the surface of the acumen and microorganisms strongly colocalize with trichomes, as can be observed in panel **(B)** a magnification of two trichomes in panel **(A)**, as well as in panel **(C)**, showing another pair of trichomes densely colonized by microorganisms. Scale bars represent 20 and 10 μm in panels **(A)** and **(B,C)**, respectively.

**FIGURE 3:**
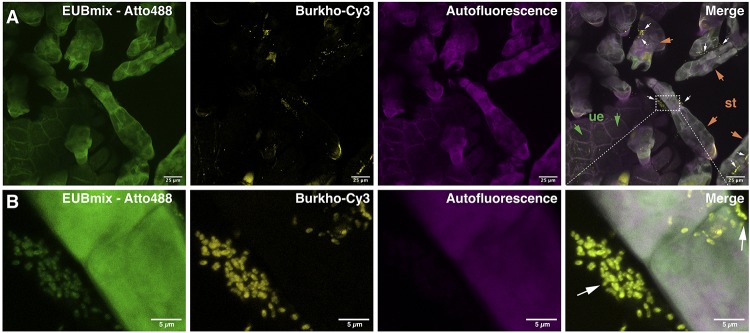
Visualization of clusters of *Burkholderia sensu lato* spp. associated with secretory trichomes on leaf acumens of *Dioscorea bulbifera* (potato yam) by DOPE-FISH/CLSM microscopy. EUBmix and Burkho probes were applied onto leaf acumens sequentially and the upper surface of the acumen, which is densely covered with secretory trichomes, was visualized **(A)**. *Burkholderia s.l.* spp. (white arrows), labeled with both EUBmix (green channel) and Burkho probes (yellow channel), can be observed clustering on the sides of, or in between, secretory trichomes (st, orange arrows) emerging from the upper epidermis (ue, green arrows) of the leaf acumen. In panel **(B)** a high-resolution image of a cluster on the side of a trichome can be seen, all bacterial cells shown are labeled with the *Burkholderia s.l.* specific probe.

To demonstrate that bacteria are indeed quite abundant on the surface of the *D. bulbifera* leaf acumen we analyzed parts of the leaves by means of confocal microscopy. In order to visualize bacteria, we stained nucleic acids with a SYBR^®^ Green general DNA stain and made use of the plant autofluorescence signature to image various plant structures in the background. In contrast to the leaf lamina the leaf acumen is densely covered with trichomes that emit a characteristic autofluorescence signal between 571 and 655 nm when excited at 550−555 nm (Figure 2A). This signal can nicely be separated from chlorophyll autofluorescence (em 674−716 nm) as well as that of other plant surface specific structures (cuticle etc.), which suggests the presence of certain compounds within the cell wall or the excretions of the trichome causing the signal. We used the specificity of the signal to reduce the plant associated background while still visualizing the trichomes. Bacteria can indeed be observed in exceptionally high numbers (Figure 2). They consistently occur within large clusters on the surface of trichomes (Figures 2B,C). Interestingly, these clusters could already be observed at very early stages in the development of the leaf, in fact, at a stage where the leaf hardly consists of anything but the acumen. Naturally, this led to the question of the identity of the bacteria. To see whether *Burkholderia s.l.* species were among the trichome associated microbiota we conducted fluorescence *in situ* hybridization (FISH) experiments using double labeling of oligonucleotide probes (DOPE-FISH) coupled with confocal laser-scanning microscopy (CLSM). Bacteria were labeled with an Atto488 coupled mix of EUB probes (EUBmix) and members of the *Burkholderia s.l*. species cluster were labeled with the Cy3 tagged probe Burkho. Indeed, we could show that the bacteria that form clusters on or in between secretory trichomes are almost exclusively *Burkholderia s.l.* spp. (Figures 3A,B). We also observed other bacteria-like structures after coupling of DOPE-FISH using the Burkho probe and SYBR^®^ Green staining (Supplementary Figure 3). However, these were never observed as clusters on trichomes and, even outside of clusters, the vast majority of cells emitted a Cy3 signal, indicating they, too, were *Burkholderia s.l.* species.

### Strain Msb3 Represents a Novel *Paraburkholderia* Species

To further analyze the role of strain Msb3 in its occupied niche and its phylogenetic affiliation we sequenced and assembled its genome. Average nucleotide identity (ANI) values were calculated for selected genome pairs from a dataset of 15 genomes of closely related taxa (see Supplementary Information), making use of the JSpecies Web Server (Richter et al., 2016; Table 2 and Supplementary Table 1). The closest relative of strain Msb3 is *Burkholderia* sp. Ch1-1 with an ANIb value of 98.89% and an alignment fraction (AF) of 0.87. According to the 95% threshold for species delineation (Kim et al., 2014) this classifies them as the same species. Strain Ch1-1 has not been described yet and information concerning its lifestyle is scarce (NCBI BioProject: PRJNA224116) nor is it available in public strain collections. *P. xenovorans* is the closest classified relative of strain Msb3 and its type strain shares an ANIb value of 93.54% with (AF = 0.78) with strain Msb3, indicating that they are distinct species (Kim et al., 2014). The type strains of *P. aromaticivorans* and *P. phytofirmans* are also closely related to strain Msb3 but fall far below the threshold with ANIb values of 89% and 87%, respectively. These results indicate that strain Msb3 represents a novel species.

**TABLE 2:** ANIb values and percentage of aligned sequences between selected genomes and the genome of strain Msb3.

***x***	***x* to strain Msb3**	**Strain Msb3 to *x***
*Burkholderia* sp. Ch1-1	98.59 [84.07]	98.89 [87.49]
*Paraburkholderia xenovorans* LB400 [T]	92.23 [71.01]	93.54 [78.62]
*Paraburkholderia aromaticivorans* BN5 [T]	89.03 [67.35]	89.29 [71.24]
*Paraburkholderia phytofirmans* PsJN [T]	86.85 [65.82]	87.07 [64.36]
*Paraburkholderia terricola* LMG 20594 [T]	84.71 [58.16]	84.01 [53.56]
*Paraburkholderia sediminicola* LMG 24238 [T]	84.61 [58.38]	84.01 [53.52]
*Paraburkholderia bryophila* LMG 23644 [T]	84.17 [57.87]	83.98 [56.44]
*Paraburkholderia fungorum* NBRC 102489 [T]	83.36 [55.39]	83.57 [57.28]
*Burkholderia* sp. ST111	83.31 [56.58]	83.98 [57.89]
*Paraburkholderia aspalathi* LMG 27731 [T]	83.10 [52.58]	84.15 [59.71]
*Paraburkholderia insulsa* LMG 28183 [T]	82.86 [52.46]	83.39 [59.23]
*Paraburkholderia rhynchosiae* WSM 3937 [T]	82.60 [53.69]	82.57 [52.28]
*Paraburkholderia caledonica* NBRC 102488 [T]	82.09 [58.91]	81.92 [52.90]
*Paraburkholderia kirstenboschensis* KB15 [T]	81.94 [53.94]	82.24 [51.56]

### Genome of Strain Msb3

#### General Genome Properties

**FIGURE 4:**
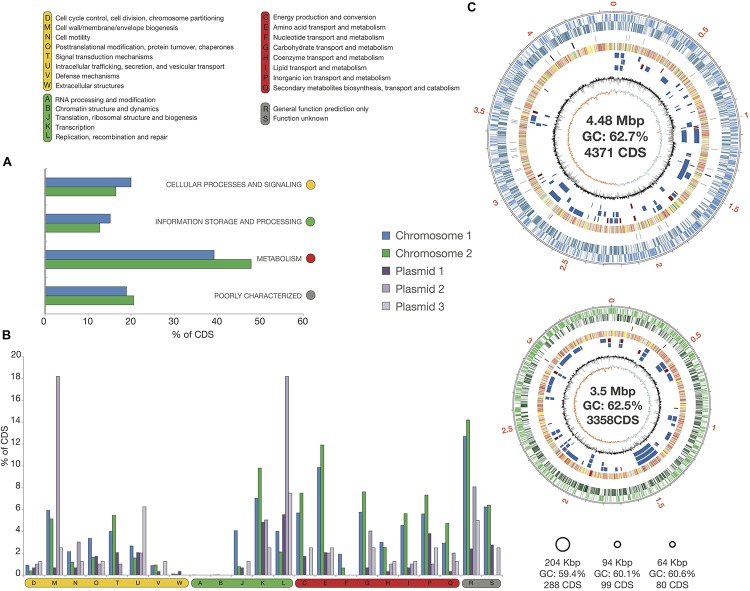
Circular Replicons and COG distribution in the Msb3 Genome. **(A)** Classification of CDS into functional categories based on clusters of orthologous groups of proteins (COGs) on the two chromosomes. **(B)** Functional distribution over the five replicons, based on the COG classification. Values in panels **(A,B)** are shown as the percentage of genes on each replicon belonging to each COG. **(C)** Representation of circular replicons found in the genome of strain Msb3. Each replicon is sorrounded by a black circle, indicating the size of the contig in Mpb but also reflecting its relative size compared to other replicons. The two chromosomes were investigated in more detail. The meaning of each circle, starting from the outside, is as follows: circle 1 displays contig size in Mpb; circle 2 and 3 display CDS on (+) and (–) strand, respectively; circle 4 shows the location of RNAs on each chromosome, rRNAs in red and tRNAs in black; circle 5 carries COG functional classifications of each CDS classified within a COG; circle 6–8 show genomic islands of strain Msb3 when compared to its closest relatives, where circle 6 represents PsJN, circle 7 represents LB400 and circle 8 Ch1-1: a blue box is a genomic island with more than 40% of all CDS within the region absent from the relatives’ genome and a red box an island with 100% of CDS absent from the genome of the close relative. Circle 9 displays a histogram of the G + C content (mol%) calculated per gene, relative to the average of the whole genome (62.55 mol%). Circle 10 shows cumulative G + C skew calculations in steps of 1000 bp, relative to 0.

The genome of strain Msb3 has a size of 8.35 Mbp, harboring 8,199 coding sequences (CDS) distributed over five circular replicons – two chromosomes and three plasmids – designated C1 (4.48 Mbp), C2 (3.5 Mbp), P1 (204 Kbp), P2 (94 Kbp), and P3 (64 Kbp) in agreement with the described *P. xenovorans* strain LB400 (henceforth LB400) replicon structure (Chain et al., 2006). Origins of replication were determined based on the presence of genes involved in DNA replication close to the predicted origin by G + C skew analysis (Figure 4). The total G + C% of the genome of strain Msb3 is 62.52%. Summed genomic properties are usual for *Paraburkholderia* species, shown in Table 3 and details concerning plasmids as well as a comparison of replicon specific clusters of orthologous groups of proteins (COG) classification patterns and biases toward certain groups can be observed in Figure 4 and are further discussed in Supplementary Information.

**TABLE 3:** General properties of the strain Msb3 genome assembly.

	**Total**	**C1**	**C2**	**P1**	**P2**	**P3**
Average GC% content	62.52	62.7	62.55	59.63	60.12	60.56
Size (bp)	8,347,503	4,479,997	3,505,044	204,567	93,611	64,284
Nr. predicted CDS	8,199	4,374	3,358	288	99	80
% protein coding density	87.81	87.49	88.3	86.55	83.66	84.64
rRNA operons	6	3	3	0	0	0
tRNAs	63	55	7	1	0	0
Contigs	5	1	1	1	1	1
Circular		+	+	+	+	−
Completeness	99.92%					

#### Primary Metabolism

Strain Msb3 carries a large genome shaped for versatility. A total of 3,378 CDS classified within COGs related to metabolic processes present a large repertoire of genes executing various metabolic functions. Through analysis of carbohydrate metabolism and transport we can infer a heterotrophic or potentially mixotrophic growth mode for strain Msb3. A detailed reconstruction of strain Msb3s primary metabolism can be found in Supplementary Information. Noteworthy is the identification of several putative TRAP-family dicarboxylic acid transporters, suggesting that C4 dicarboxylic acids could be used for energy production through enzymatic carboxylate degradation in the tricarboxylic acid (TCA) cycle. Interestingly, specific TRAP transporters were found among genes shared only by strain Msb3 and *P. phytofirmans* strain PsJN (henceforth PsJN) but not LB400, indicating that adaptations to specific environmental niches may have reshaped central carbon acquisition strategies. Phenotypic assays were conducted showing aerobic growth on malate and citrate. Dicarboxylic acids are among the preferred substrates for growth and may provide energy and a source of carbon for growth *in planta*.

Nitrogen metabolism encompasses most features usually found within most relatives of strain Msb3. The presence of an entire *nif* nitrogen fixation gene cluster located on C2 is worth mentioning as this could enable the strain to perform BNF, a feature that is lacking from the PsJN genome. More information can be retrieved from Supplementary Information and Supplementary Table 2.

#### Secondary Metabolism

On C2 we identified a non-ribosomal peptide synthetase (NRPS) consisting of a two-protein core cluster, an “amino acid adenylation enzyme/thioester reductase family protein” as well as a “non-ribosomal peptide synthase/amino acid adenylation enzyme” predicted by antiSMASH, and a border cluster containing an ABC-superfamily cyclic peptide transporter as well as all necessary transport system components. Gene clusters predicted to produce some bacteriocins were detected on both chromosomes, including one responsible for linocin-M18 production.

Terpenoid biosynthesis is also prominently represented by three protein clusters, two of which encode a putative squalene synthase (SQS), responsible for the catalytic reduction of two farnesyl pyrophosphate molecules to squalene through the consumption of NADPH. Squalene is a parent molecule of cyclic triterpenoids. Appropriately, we found a *hpn* (hopanoid biosynthesis) operon (*hpnBCDEN, shc, nifB*). It encodes enzymes for hopanoid biosynthesis, structural modifications and transport. *Squalene-hopene cyclase* (*shc*) can synthesize cyclic triterpenoids directly from squalene. Resulting hopanoid lipids are thought to provide membrane stability and to mediate stress resistance under low pH and high osmotic pressure (Belin et al., 2018) and, interestingly, many hopanoid-producing bacteria are capable of nitrogen fixation (Berry et al., 1993; Belin et al., 2018) or live in plant-associated environments (Ricci et al., 2014).

Finally, we identified a type I polyketide synthase (T1PKS) cluster on P2. It is part of a larger gene cluster associated with capsule polysaccharide production and export. The synthesis of many complex polyketide antibiotics or immunosuppressants in bacteria is catalyzed by such multimodular PKSs.

#### Genetic Factors Defining the Specific Niche of Strain Msb3

Macromolecular systems in the genome of Msb3 were predicted using the MacSyFinder tool (Abby et al., 2014) using the models from Abby et al. (2016) for the identification of protein secretion systems and closely related macromolecular systems in bacterial genomes. 15 loci encoding 11 different systems were identified in the genome of strain Msb3. Apart from a complete flagellum and two complete Tad pilus loci it harbors complete T1SS (3x), T2SS, T3SS, T5aSS, T5bSS (3x) and T5cSS (2x). Additionally, an incomplete T6SS was predicted, located on C2, but missing three necessary genes: *tssA*, *tssI* and *evpJ*. Upon closer inspection of the locus, however, a TssA homolog was identified carrying the TssA characteristic N-terminal ImpA domain (PFAM: PF06812) with an unusual C-terminal extension. We also identified two VgrG (=TssI) orthologs, encoded away from the main locus on C1. Furthermore, the functioning of the T6SS is not always dependent on EvpJ as demonstrated in *Edwardsiella tarda* by Zheng and Leung (2007). Therefore, the T6SS can be considered functional, remaining to be experimentally confirmed. Interestingly, the complete T6SS is among those elements shared between strain Msb3 and PsJN, but absent in LB400 (Supplementary Information and Supplementary Figure 4).

The expression of motility genes is under strict regulation and is mediated through sensing of environmental stimuli (Osterman et al., 2015). The Msb3 genome encodes 37 genes involved in chemotaxis (Che protein response regulators). This, together with quorum sensing mechanisms like *luxR*-like and N-acyl-homoserine lactone (AHL) based systems found in the genome could enable the strain to sense a set of environmental stimuli and to adapt to them. Motility genes, together with the presence of genes encoding for plant cell wall-degrading enzymes (cellulases and endoglucanases), could help to explain the systemic plant colonization by strain Msb3 in *D. bulbifera.*

Key adaptations for the colonization of eukaryotic hosts are mechanisms to cope with the consequences of host defense mechanisms. Reactive oxygen species (ROS) detoxification as well as xenobiotic degradation are common strategies of plant endophytes. The Msb3 genome shows a range of detoxification mechanisms including various enzymes necessary to cope with oxidative stress (catalases peroxidases), heavy metal and drug efflux systems and the degradation of organic substances (Supplementary Table 3). Additionally, multiple glutathion-S-transferases (GSTs) (17×) confer defense against both acute and chronic toxicities of electrophiles and reactive oxygen/nitrogen species, which includes processes such as the biodegradation of xenobiotics and antimicrobial drug resistance (Hayes et al., 2005; Allocati et al., 2009).

#### Genes Involved in Plant Growth Promotion

Activity of the enzyme ACC-deaminase (*acdS*) has been shown to significantly contribute to plant growth (Shah et al., 1998; Penrose and Glick, 2003) and represents the best way for plant growth promotion (PGP) besides IAA production (Olanrewaju et al., 2017). The presence of both IAA biosynthesis through indole-3-acetonitrile nitrilase, as well as ACC-deaminase (*acdS*) genes is a strong indication for the important role these genes play in the plant-associated lifestyle strategies of this strain.

**FIGURE 5:**
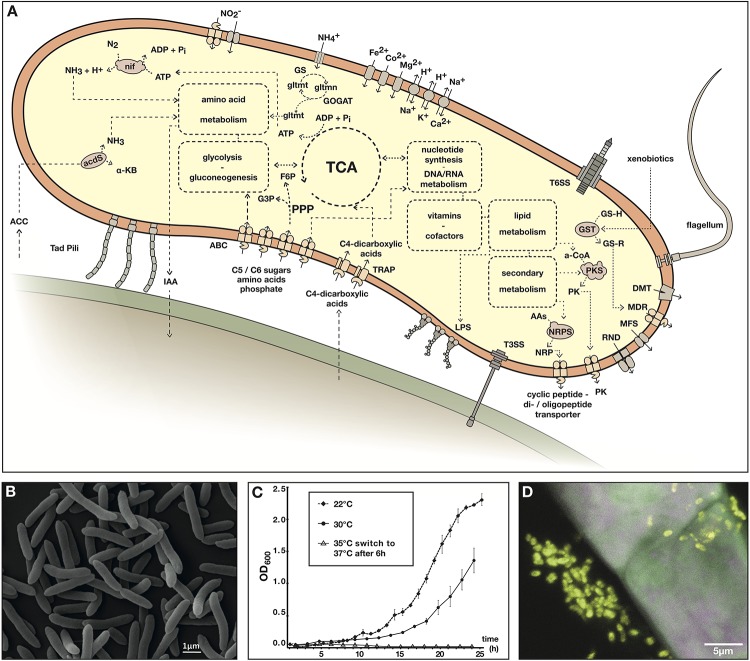
*Paraburkholderia* sp. strain Msb3. **(A)** Metabolic reconstruction based on the genome sequence of strain Msb3. Depicted are processes discussed in the main text that are thought to be relevant in the occupation of plants as a specific niche. The cartoon representation includes core metabolisms that are insinuated through dashed boxes and circles. Dashed lines and arrows indicate connectivity of pathways or suggest transport of substances. Full arrows indicate translocation across membranes through transporters. Pathways, transporters, protein complexes and substances are labeled with abbreviations as follows: ACC, 1-aminocyclopropane-1-carboxylic acid; acdS, ACC-deaminase; α-KB, α-ketobutyrate; GS, glutamine synthetase; GOGAT, glutamine oxoglutarate aminotransferase; gltmn, glutamine; gltmt, glutamate; IAA, indole-3-acetic acid; ABC, ATP-binding cassette transporter; PPP, pentose phosphate pathway; G3P, glyceraldehyde 3-phosphate; F6P, fructose 6-phosphate; TCA, tricarboxylic acid cycle; TRAP, tripartite ATP-independent periplasmic transporters; LPS, lipopolysaccharide; T3SS, type III secretion system; T6SS, type VI secretion system; GSH, glutathione; -R, rest; GST, glutathione S-transferases; t1PKS, type I polyketide synthase; PK, polyketide; AAs, amino acids; NRPS, non-ribosomal peptide synthetase; NRP, non-ribosomal peptide; RND, resistance-nodulation-division family transporters; MFS, major facilitator superfamily; MDR, multi-drug resistance ABC transporter; DMT, drug/metabolite transporter superfamily. **(B)** Scanning electron micrograph of strain Msb3 in pure culture. **(C)** Growth curves of strain Msb3 at different temperatures in liquid TSB medium, growth rates decline with rising temperature and at 37°C growth is inhibited. OD_600_ per timepoint and temperature was measured with biological triplicates and every single measurement was performed in technical triplicates. Means of technical triplicates functioned as individual values, which were used to calculate averages and standard deviations **(D)** CLSM image overlay of a FISH experiment performed on a *D. bulbifera* leaf acumen, displaying a single secretory trichome on the surface of the acumen. Bacteria have been labeled with EUBmix-ATTO488 (green signal) and *Burkholderia s.l.* species with Burkho-Cy3 (yellow signal). Plant autofluorescence was visualized in magenta. *Burkholderia s.l.* species appear bright yellow in the overlay, whereas plant structures appear grayish magenta.

Another mechanism by which plant associated bacteria can stimulate plant growth is by supplying nutrients, most notably, nitrogen. As mentioned above strain Msb3 carries a complete nitrogenase gene cluster (*nifABZSHDKENXQ*) on C2, theoretically enabling the strain to perform BNF. This may contribute directly or indirectly to plant growth and offers a powerful tool for mutualistic plant-microbe interactions. In Figure 5A we present a metabolic reconstruction of strain Msb3, based on its genome sequence, summarizing our findings.

### Physico-Chemical Characterization of Strain Msb3

We phenotypically examined strain Msb3 (Figure 5B) in respect to its temperature growth range, its resistance and susceptibility to various antibiotics as well as its preferred carbon sources. The strain grows up to a temperature of 35°C with optimal growth observed between 22°C and 28°C. Growth on rich medium is inhibited between 36°C and 37°C. A clear growth rate reduction was observed at temperatures between 30°C and 35°C (Figure 5C).

The preferred carbon sources of strain Msb3 are simple C4 sugars. The strain can grow in minimal medium that contains glucose as well as malate and citrate as sole carbon sources, respectively. At an equimolar concentration growth on malate is more efficient than growth on glucose.

Strain Msb3 is resistant to several commonly used antibiotics including ampicillin (50−100 μg/mL), cefalexin (10−40 μg/mL), and chloramphenicol (25 μg/mL). It is, however, sensitive to standard inhibitory concentrations of carbenicillin, streptomycin as well as kanamycin.

### Strain Msb3 Promotes Plant Growth in Tomato

**FIGURE 6:**
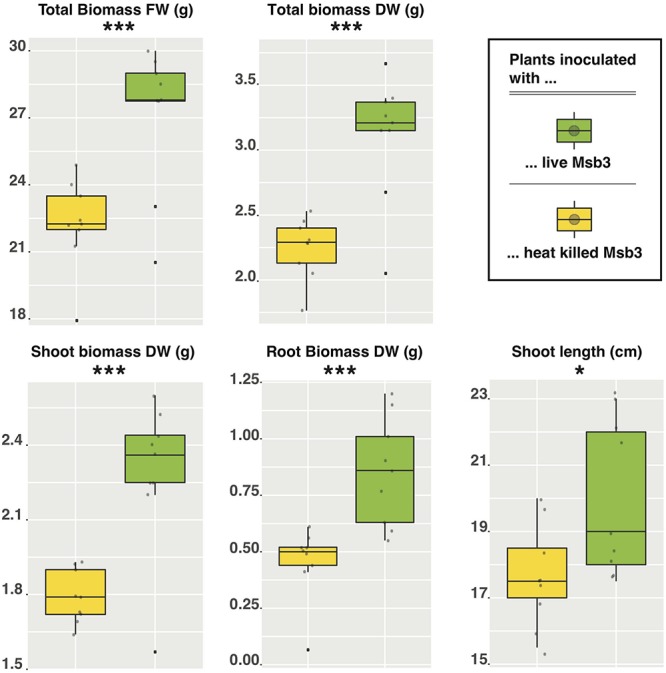
Box-plots of growth promotion assays in tomato using strain Msb3 as an inoculant. Individual tomato plants (*n* = 9) were inoculated with either live (green boxes) or heat killed (yellow boxes) cells of strain Msb3 and plant growth was compared after 34 DPI. Depicted here are comparisons between total biomass FW and DW as well as the respective contribution of shoot and root (in DW). Additionally, the shoot length was compared. Statistical significance was tested via two tailed Student’s *t*-tests.

To experimentally validate our hypotheses concerning the PGP potential of strain Msb3 we monitored the growth of tomato (*Solanum lycopersicum* L., cv. Moneymaker) seedlings inoculated with the strain. Nine biological replicates of 14-day old seedlings grown from surface disinfected seeds were inoculated with bacteria by dipping into bacterial suspension (OD = 10, 0.5−5 × 10^9^ CFUs) and were subsequently raised in sterilized soil under controlled conditions. Controls were treated with dead bacteria. 34 days post-inoculation (DPI) the plants were harvested and shoot length as well as root- and shoot-biomass were determined. We observed a highly significant effect: seedlings inoculated with live bacteria were significantly taller as well as heavier than their control counterparts (Figure 6). This effect was highly significant for all forms of biomass: Δ total biomass FW (*P* = 0.00074); Δ shoot biomass FW (*P* = 0.0073); Δ root biomass FW (*P* = 0.00067); Δ shoot biomass DW (*P* = 0.0003); Δ root biomass DW (*P* = 0.0008); Δ total biomass DW (*P* = 0.00008). Shoot length was also significantly influenced (*P* = 0.02253) by the treatment. Root biomass was influenced most heavily with an average increase of 44% (FW) and 87% (DW) compared to the control, followed by shoot biomass with an increase of 14.5% (FW) and 28% (DW), leading to a total difference of roughly 22% (FW) and 40% (DW). We subsequently tried to inoculate just the soil around the seedling and, despite the small number of biological replicates (three), retained a comparable and significant effect: 36% increase (*P* = 0.033) in root biomass. Re-cultivation of strain Msb3 from roots of inoculated tomato seedlings and plants grown in inoculated soil was successful, indicating that the strain successfully invaded the tomato rhizosphere to promote plant growth.

### Ecological and Phylogenetic Boundaries in *Burkholderia s.l.*

**FIGURE 7:**
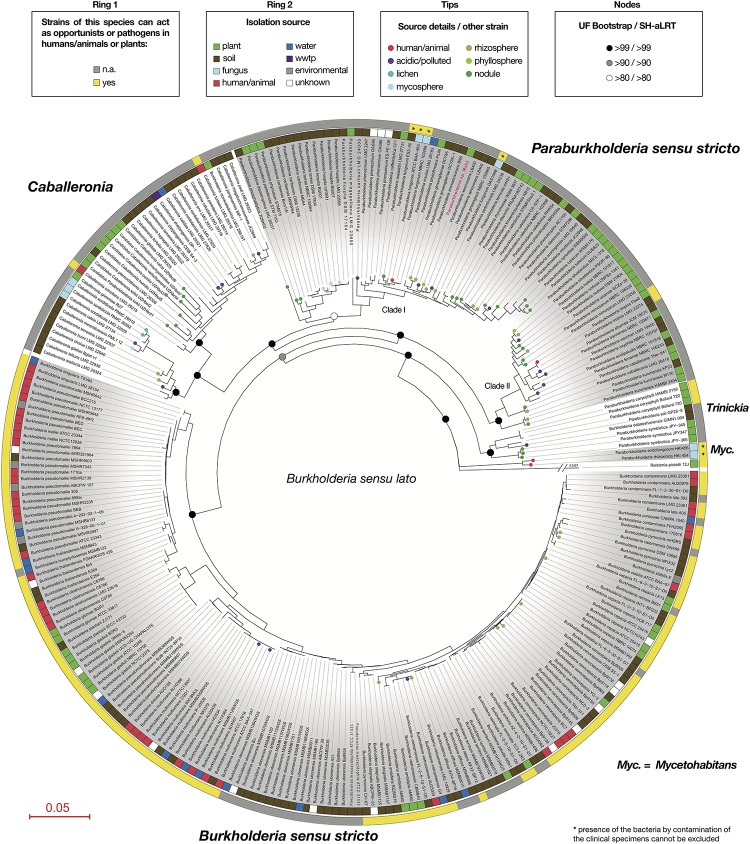
Ecological data concerning *Burkholderia sensu lato* species within a robust phylogenetic framework. A phylogenetic tree for the genus *Burkholderia sensu lato* based on the concatenated alignment of 54 ribosomal proteins. Species and strain names represent those used in the Bacterial Isolate Genome Sequence Database (BIGSdb), except for strain Msb3, which is marked in pink and carries the species name proposed in this study. The most recent genus names validly published for specific groups, distinguished through gray and white shading, are shown outside of the first circle. The first circle indicates whether one or more pathogenic strains (plant or animal) were found within a bacterial species. If there was evidence, all strains carrying the same species name were marked yellow. The second circle shows the primary isolation source of every strain included in the analysis, using a color code explained in the legend. Annotations on the tree include small circles on branch tips, showing additional details about the primary isolation source or the isolation source of strains from the same species not included in the analysis. Octagons on nodes display values for ultrafast (UF) bootstrap and approximate likelihood ratio test (aLRT) calculations with the non-parametric SH correction (SH-aLRT) in the spirit of the Shimodaira–Hasegawa (SH) algorithm. Branch length is given as amino-acid substitutions per site. *Ralstonia picketti* strain 12J was used as an outgroup to set the root of the tree.

We realized there were discrepancies between views concerning generic as well as ecological boundaries in *Burkholderia s.l*. In order to derive useful information concerning the lifestyle of strain Msb3 in respect to dangers associated to its application we set out to review current hypotheses concerning the prevalence of opportunism and pathogenicity within different clades affiliated with *Burkholderia s.l*. We generated a robust phylogenetic tree (Figure 7) using 54 concatenated ribosomal proteins containing all strains within *Burkholderia s.l.* that were available for PubMLST in the BIGSdb (Jolley et al., 2018) and extracted the sequences from a set of selected other *Burkholderiaceae*. In an extensive literature review we retrieved information about all strains, including isolation source, details about the isolation as well as information about strains not represented in the tree, but with relevant isolation sources, differing from the represented strains’ source (Supplementary Table 4). We inferred whether a species, represented by all its strains, could be considered opportunistic/pathogenic or not. Our analysis shows that the proposed ecological boundaries within *Burkholderia s.l*. are not supported: the plant-beneficial and environmental (PBE) *Burkholderia* species cluster is now comprised of five distinct genera: *Paraburkholderia s.s.*; *Caballeronia*; *Robbsia*; *Trinickia* and *Mycetohabitans*. Every single one of these genera harbors at least one species that can act as an opportunistic pathogen to either plants or humans (Figure 7) and in total we have identified at least six species within the group with connections to disease and clinical environments. *Paraburkholderia s.s.* forms two large clusters in our reconstruction, henceforth referred to as Clade I and Clade II *Paraburkholderia s.s*. Strain Msb3 branches within Clade I *Paraburkholderia s.s.*, which contains the opportunistic human colonizer *P. fungorum*.

## Discussion

As the plant phyllosphere is an underrepresented target of microbiological studies we set out to discover novel forms of associations between *Burkholderia s.l*. species and their plant hosts in this habitat. Inspired by the occurrence of the “leaf nodule symbiosis” in *Dioscorea sansibarensis* (Carlier et al., 2017; Pinto-Carbo et al., 2018; De Meyer et al., 2019) we investigated the prevalence of *Burkholderia s.l.* species in the plant family *Dioscoreaceae*. Indeed, we detected and isolated the novel *Paraburkholderia* strain Msb3 from leaf acumens of *Dioscorea bulbifera*. In our efforts to describe the strain, its ecology and its potential, we conducted several targeted experiments to gain insights into (a) its role on its host plant *D. bulbifera*, (b) its genetic and metabolic repertoire, (c) its potential for agricultural application in promoting plant growth and (d) its phylogenetic position and the potential of it and its close relatives for pathogenicity.

*Paraburkholderia* spp. generally display an array of desirable properties that could be exploited for uses in agriculture that are matched by few other groups of bacteria. To illustrate: *P. xenovorans* strain LB400 can fix nitrogen and has the ability to degrade polychlorinated biphenyl (PCB) and could be used for bioremediation of contaminated soils. *P. phytofirmans* strain PsJN is an endophytic plant growth promoting organism with an extraordinary host range (Frommel et al., 1991; Sessitsch et al., 2005; Da et al., 2012; Mitter et al., 2013) and could be used for large scale production of biofertilizers. Some *Paraburkholderia* spp. have the ability to nodulate legumes and symbiotically fix nitrogen, unusual among *Betaproteobacteria*, therefore called “β-rhizobia” (Moulin et al., 2001; Chen et al., 2003). The tendency of *Paraburkholderia* to engage in facultative symbiotic relationships with plants is unequivocal and, unlike leaf nodulating *Caballeronia* species, the degree of loose-intimacy allows us to culture and study them and to potentially transfer these traits to non-original host plants. To our best knowledge, this is the first report of a *Paraburkholderia* strain flourishing in the phyllosphere of its host plant and it is also the first implication of a strain of the genus in a facultative symbiotic relationship with a yam family plant. It should be noted that here the broad definition of symbiosis is used, as the outcome of the interaction for both parties is not fully elucidated. This definition includes mutualistic, as well as commensal and parasitic associations and generally refers to any type of a close, prolonged interaction between two different organisms.

We consistently detected the strains molecular signature in plant DNA extracts of *D. bulbifera* over a period of 3 years, representing three complete growth cycles of the perennial vine. We also retrieved high numbers of cells from crude leaf acumen extracts in two independent cultivation trials, a year apart, from the same plant individual and detected the strains 16S rDNA in several individuals that had been reproduced clonally through underground tubers. We hypothesized about the role of strain Msb3 among the leaf microbiota on *D. bulbifera* and about the actual abundance and position on the plant. Using the described amplicon sequencing approach, we were able to show that strain Msb3 indeed localizes at leaf acumens. Since surface sterilization of the acumen lead to a significant reduction of bacterial reads, heavily influencing the relative abundance of strain Msb3, the organism does not seem to reside inside the plant but rather dwells on the acumen surface instead. Indeed, microscopic analysis showed that bacteria are associated to trichomes on the acumen (Figures 2, 3, 5D). Further visual exploration of secretory trichomes on the leaf acumen via FISH using a genus specific probe lead to the identification of clusters of bacteria affiliated with *Burkholderia s.l*., colonizing the sides of trichomes (Figure 3), further supporting our hypothesis. We generally found few other bacteria. This may be an artifact due to weak signal intensity compared to the plant background and fast bleaching of Atto488, yet, these findings were supported by other experiments using a general DNA stain instead of the EUBmix (Supplementary Figure 3). Together with the community profiling data these results provide strong evidence that strain Msb3 plays a key role in its niche, as it clearly dominates this bacterial community.

The observed association could offer an intriguing perspective on the evolution of the “leaf nodule symbiosis” in *Dioscoreaceae*. *D. sansibarensis* is considered the only species of the monocot family, in which nodule formation occurs (Carlier et al., 2017; Pinto-Carbo et al., 2018; De Meyer et al., 2019), however, the genomic similarity between our strain and *O. dioscoreae* (Carlier et al., 2017), their position and abundance on the leaf and the role of secretory trichomes (Miller and Reporter, 1987; Pinto-Carbo et al., 2018) as well as the close phylogenetic relationship of both hosts (Viruel et al., 2018) and microbes (Carlier et al., 2017) are indicative for conserved structures mediating these associations. Both symbionts are *bona fide* epiphytes for at least part of their life (Carlier et al., 2017) and form clusters on secretory trichomes on the leaf acumen (Miller and Reporter, 1987) of their true yam host plant. In light of the absence of nodules in *D. bulbifera* our findings could be interpreted as an early stage in the development of such a structure. As we have not actually elucidated the mechanics of the interaction, or the transmission of the symbiont, we refrain from defining it as the discovery of a novel form of the leaf symbiosis. Nevertheless, we believe there are striking parallels between this association and the leaf symbiosis in *D. sansibarensis*. The herein described genomic properties resemble many of those found and upregulated *in planta* in *O. dioscoreae* (De Meyer et al., 2019): a complete T6SS, a NRPS, polyketide synthesis, prominent organic acid and TCA intermediate metabolism, ammonia assimilation and a functional GS/GOGAT pathway, ROS detoxification mechanisms and an ACC deaminase.

Strain Msb3 thereby displays most properties associated with plant associated and endophytic lifestyles and offers some intriguing strategies for PGP and interaction with various hosts. The gene repertoire associated with host colonization, in particular the abundance of macromolecular systems, is indicative of a large host range, comparable to that of PsJN. Indeed, the T6SS is absent in most close relatives of strain Msb3, but conserved in both this strain and PsJN, underlining its potential importance in successful plant colonization. PGP mechanisms include plant hormone regulation strategies, i.e., ACC deamination and IAA synthesis. Growth promotion through PsJN is thought to be mediated this way (Sessitsch et al., 2005) and the genes are absent in soil dwelling LB400. The combination of these genes with a nitrogenase gene cluster is remarkable. Although nitrogenase enzymes are extremely sensitive to oxygen (Gallon, 1992) *in vitro* diazotrophy has been shown in many plant-associated, non-legume-nodulating *Paraburkholderia* species, including *P. unamae, P. tropica, P. silvatlantica, P. xenovorans, P. vietnamiensis* and *P. kururiensis* (Martinez-Aguilar et al., 2008; Suarez-Moreno et al., 2012). The rare consolidation of these features, BNF and plant hormone regulation, constitutes a powerful tool for the production of potent biofertilizers.

The exact role of strain Msb3 in the association to *D. bulbifera* remains unclear. Secondary metabolism may well play an important role. It does in the association of *O. dioscoreae* to *D. sansibarensis* (De Meyer et al., 2019) and similarly, strain Msb3 carries gene clusters for the production of a non-ribosomal peptide and a polyketide hitherto unknown. So far, we were not able to produce axenic *D. bulbifera* plants, stalling our efforts to further investigate it and low bacterial biomass compared to plant biomass exacerbates applications such as analysis of the leaf meta-transcriptome. We do know, however, that strain Msb3 strongly promotes plant growth in another plant, i.e., tomato. We tested its potential for PGP under semi-sterile conditions and observed a strong and highly significant effect on plant biomass upon inoculation of seedlings or soil. Here, growth promotion is most likely mediated through colonization of the tomato rhizosphere. The fact that strain Msb3 confers PGP to hosts beside *D. bulbifera* suggests a large host range and shows its ability to quickly adapt to various ecological niches. These traits make it an ideal candidate for investigation in respect to agricultural applications. This is underlined by the fact that it lacks the ability to survive at temperatures higher than 36°C, making colonization of mammalian hosts unlikely.

Nevertheless, the application of PGPB and bioagents in the environment is connected to several risks that need to be understood and eliminated before administration. The possibility of releasing pathogens (to humans but also other vertebrates and plants) into the environment and possibly into the food chain must be averted at all cost. *Paraburkholderia* are often viewed as unproblematic and even beneficial, as their phylogenetic difference from pathogenic and opportunistic *Burkholderia s.s*. is undisputed and its isolates almost exclusively come from environmental samples, usually from soil and plants (Suarez-Moreno et al., 2012; Angus et al., 2014; Sawana et al., 2014; Estrada-de los Santos et al., 2015). However, this is subject to a controversial debate within the community (Estrada-de los Santos et al., 2015; Eberl and Vandamme, 2016; Vandamme et al., 2017; Dobritsa and Samadpour, 2019). To clear out misconceptions and to tackle safety concerns we reevaluated phylogenetic relationships within *Burkholderia s.l.* in a refined phylogeny using data from almost 280 different isolates and mapped ecological data points, retrieved from extensive literature review, onto our robust phylogenetic framework. Our strain level analysis clearly shows that the ecological characterization along phylogenetic boundaries within *Burkholderia s.l.* is by no means an accurate way to classify environmental strains as unproblematic. The five genera originating from the PBE *Burkholderia* species cluster contain opportunists: (1) *Caballeronia* (Dobritsa and Samadpour, 2016) contains two species that are potentially opportunistic human colonizers: *Caballeronia turbans* and *Caballeronia concitans* were both isolated from human clinical samples, i.e., blood, pleural fluid and lung tissue (Peeters et al., 2016). (2) *Robbsia* (Lopes-Santos et al., 2017; Oren and Garrity, 2017) is represented by a single species, *Robbsia andropogonis*, which was isolated from stripe disease in sorghum (Smith, 1911) and causes leaf spots, streaks, or stripes in a wide host range. The plant pathogen is, however, no longer considered part of the *Burkholderia s.l.* species complex (Lopes-Santos et al., 2017) and has therefore not been included in our tree. (3) *Trinickia* (Estrada-de Los Santos et al., 2018; Oren and Garrity, 2018) contains the known phytopathogen *T. caryophylli*, which is the causative agent of bacterial wilt and root rot of carnations (Burkholder, 1942). (4) *Mycetohabitans* is comprised of two species, *M. endofungorum* and *M. rhizoxinica* (Partida-Martinez et al., 2007). While Estrada-de Los Santos et al. (2018) claim that these species are not likely to be pathogenic a study by Gee et al. (2011) shows that strains of both species could be isolated from clinical samples, including human blood and wounds. Although strong statements about their actual pathogenicity are inappropriate, due to a lack of information about the clinical background of these isolates, these species should be carefully investigated and opportunistic behavior should be assumed. (5) A similar case can be observed in the emended genus *Paraburkholderia* (here *s*.*s*.). Some isolates of *P. fungorum* originated from human or mice clinical samples (Coenye et al., 2001), including cystic fibrosis patients (Coenye et al., 2002) and a case of bacteremia with *P. fungorum* with clinical features of septic arthritis has been observed (Gerrits et al., 2005). Additionally, *P. tropica*, a well-studied free-living nitrogen fixing plant-associated bacterium (Reis et al., 2004), has been found to colonize immunocompromised patients. The patient developed septicemia due to *P. tropica* infection of the blood (Deris et al., 2010). 16S rRNA analysis of the blood culture gave a 100% identity match to that of *P. tropica*, unambiguously demonstrating that at least one strain of this species can be considered an opportunist in immunocompromised patients.

It ought to be mentioned that most of the cases described above have been limited to very few occurrences, usually only in immunocompromised patients, suggesting that the risks they pose to healthy individuals is rather small. Nevertheless, classification of any novel isolate as unproblematic should be conducted with great care. We therefore emphasize that such classification of any species or strain within the genera investigated here, including that of Msb3, must be made on a case-by-case basis, only after careful molecular and phenotypic characterization. The need for a consensus within the community, outlining the procedures for their characterization (e.g., growth at 37°C, suitable multispecies infection scenarios, mammalian hosts, etc.) is certainly necessary.

Finally, we believe that detailed analysis of whole genomes, on a strain level rather than species level, using explicit ecological data (e.g., host range, isolation source) may help to elucidate genomic adaptations to opportunism and pathogenicity in plants or animals previously gone unnoticed. Providing genomic as well as ecological data with every description of novel strains may facilitate this process. *Paraburkholderia* sp. strain Msb3 certainly presents a promising model to study the genetic prerequisites for the development of facultative symbiotic interactions within *Burkholderia s.l.* as well as a resourceful candidate to investigate and generate biofertilizers with a broad host range.

## Data Availability Statement

Genome data from *Paraburkholderia* sp. strain Msb3 has been submitted to ENA in BioProject PRJEB33427. The whole genome sequence is available under accession numbers LR699553-LR699557. The nucleotide sequence data obtained through 16S rRNA amplicon sequencing have been submitted to the Sequence Read Archive of NCBI under BioProject number PRJNA562285.

## Author Contributions

WW and JH conceived and designed the research. JH and FS performed the bacteria isolation and the growth promotion experiments. JH performed the molecular biology experiments, genome annotation, analysis of amplicon sequencing data, and phylogenetic analyses, and wrote the manuscript with contribution from WW. SK, SG, BF, and MS performed the amplicon sequencing experiments and data preparation. JH and MB provided the microbiological and pysico-chemical data. JH, BD, and AB provided the microscopy images and performed fluorescence *in situ* hybridization experiments. JH, FS, and LF identified and collected the samples. JH, MK, and WW analyzed and interpreted the results. All authors discussed and revised the manuscript.

## Conflict of Interest

The authors declare that the research was conducted in the absence of any commercial or financial relationships that could be construed as a potential conflict of interest.

## Footnotes

1 http://www.genoscope.cns.fr/agc/microscope/home/index.php

2 http://jspecies.ribohost.com/jspeciesws/#home

3 https://gtdb.ecogenomic.org/
