# Supplementary material for: The Potato Yam Phyllosphere Ectosymbiont Paraburkholderia sp. Msb3 Is a Potent Growth Promotor in Tomato
Source: Front Microbiol. 2020 Apr 21;11:581. doi: 10.3389/fmicb.2020.00581 (PMC7186400; doi:10.3389/fmicb.2020.00581)
Supplement: Supplementary file 1 [file Data_Sheet_1.pdf]

Supplementary Material

Content:

Supplementary Information: Contains textual information useful but not imperative for the interpretation of data presented in the main manuscript as well as supplementary figures and tables.

Supplementary Figure 1: PCR images of strain Msb3 detection in leave and acumen DNA of D. bulbifera

Supplementary Figure 2: Supplementary material for amplicon sequencing data

Supplementary Figure 3: Supplementary material for fluorescence *in situ* hybridization experiments

Supplementary Figure 4: CDS classified through COGtriangles shared among PsJN, LB400 and strain Msb3

Supplementary Tables are provided as separate files that contain:

Supplementary Table 1: ANIb, ANIm and TSC matrix with close relatives of strain Msb3

Supplementary Table 2: Locus tags and annotations of CDS discussed in the document

Supplementary Table 3: List of genomic objects associated with plant association and comparison to PsJN

Supplementary Table 4: rMLST: organisms included in the analysis, their PubMLST ID and annotations derived from literature

16S rRNA phylogeny

Strain Msb3 harbors six 16S rRNA genes distributed throughout its genome, with two chromosomes harboring three copies each. One mismatch differentiates the sequences from each chromosome. Full-length 16S rRNA gene sequences from the larger chromosome share 99.3% sequence identity with P. xenovorans LB400 and 98.9% sequence identity with P. phytofirmans PsJN. While LB400 is clearly more closely related to strain Msb3, both LB400 and PsJN fall above the 98.65% 16S rRNA gene sequence similarity threshold currently used for differentiation of two species. Because of the insufficiency of SSU rRNA sequence identity for microbial species demarcation it has been replaced by the ANI (Average Nucleotide Identity) boundary value of 95%.

Amplicon sequencing details

On an Illumina MiSeq sequencing platform, we sequenced amplicon libraries that were prepared from D. bulbifera leave DNA using the primer pair 335F/769R for amplification of variable regions V3-V4 of bacterial 16 rRNA genes within a plant background. Following conditions were separated: leaves (L), surface-sterile leaves (LS), acumens (A), surface-sterile acumens (AS) and surface microbiota (-S). Sequences were analyzed using the QIIME 2 software package (Caporaso et al., 2010) and the plugin DADA2 (Callahan et al., 2016) for quality control, resulting in specific amplicon sequence variants (ASVs). A total of 2,828,470 raw reads were obtained and after quality filtering and chimera removal 1,655,557 high-quality sequences remained, which were assigned to 1,112 unique ASVs. ASVs occurring in extraction and PCR control samples as well as unassigned reads and singletons were removed, resulting in 1,653,711 partial 16S rRNA gene sequences for final analysis. To compare samples without statistical bias, subsampling with 17,702 reads was performed, reflecting the lowest read number obtained per sample.

We then compared the bacterial load between sample types according to the relative number of reads classified as order "Chloroplast" compared to others. The plant-sequence-derived ASVs were subsequently removed. We normalized usable reads by rarefying to 739 reads per sample and removed reads not prevalent in at least 5% of all samples. Rarefaction analysis indicated diversity coverage of > 99 % and consequently an adequate sampling depth for further analysis. We thereby defined the core dataset of 993 ASVs that is described in detail in the main manuscript and further visual information can be found in Supplementary Fig. 2.

General genome properties of strain Msb3

Plasmids contain neither ribosomal RNA genes nor any known essential functions, but they all carry genes for replication and partition and their overall G+C% is 1.9% to 3.5% lower than the average of the two chromosomes. This is indicative of horizontal gene transfer (HGT), supported by a complete lack of synteny with closely related strains.

Chromosome core functions were analyzed by comparison of replicon specific clusters of orthologous groups of proteins (COG) classification patterns and biases towards certain groups. The distribution of COG functions across the chromosomes is noteworthy: C1 displays a higher relative abundance of CDS involved in core cellular processes while C2 is

enriched in CDS for metabolic processes (Fig. 2a). Chain et al. (Chain et al., 2006) suggested that genomes of this group of organisms share this particular organization: the larger chromosome represents the ‘core chromosome’ conserved in core cellular functions and the smaller chromosome has a functional bias toward energy, ion and amino acid metabolism and transport as well as secondary metabolism. The smaller replicon constitutes a playground for rearrangements, which eventually leads to a specific genetic lifestyle-determining composition, indicating that genes located on C2 should be considered with priority in trying to assess the niche-specific adaptations of strain Msb3.

Plasmids in strain Msb3

Strain Msb3 carries three plasmids, none of which are shared with its closest relatives. Not a single synteny region could be identified with either LB400, PsJN nor strain Ch1-1. P1 shares several synteny regions with strains from the Burkholderia cepacia complex (Bcc), including Burkholderia cenocepacia strains and other pathogens. It should be investigated in respect to possible pathogenicity of strain Msb3; however, the closest resemblance is only 32.29 % identity to the Bcc strain TJI49 with 93 CDS in 26 syntons, most of which are hypothetical proteins of unknown function. Similarly, P2 shares several conserved regions with Bcc species with a maximum of 32 CDS with similarity to CDS found on the B. cenocepacia strain 842 chromosome 2 (NZ_CP015033) but functional annotation lacks information.

P3 shows high similarity to strains of other Paraburkholderia species: 83.75 % of all CDS located on the plasmid can be found in Paraburkholderia terrae strain BS007 and good hits are also obtained for various other Paraburkholderia species isolated from soil.

The specific function of each plasmid is unknown. In total only 35.7 % of CDS located on a plasmid are even classified into a specific COG and 20.35 % of these are classified as ‘poorly characterized’. Characterized CDS mostly fall into COG L and K (replication, recombination and repair and transcription, respectively) making up another 36.5% of CDS classified, probably involved in plasmid replication. Interesting are 18.18 % of CDS involved in ‘cell wall/membrane/envelope biogenesis’ (COG M) on P2 and 6.25 % involved in ‘intracellular trafficking, secretion, and vesicular transport’ on P3, suggesting that the plasmids indeed play a role in host associated lifestyles.

More detailed reconstruction of the primary and secondary metabolism of strain Msb3:

Carbohydrate metabolism and transport. Like its heterotrophic close relatives, strain Msb3 can theoretically oxidize a wide variety of carbon compounds. Complete glycolysis/gluconeogenesis pathways are present in the genome and explain the high growth rate (~8 generations/day at RT) of this organism under optimal conditions. A large set of ATP binding cassette (ABC) superfamily sugar transporters can mediate the uptake of C5 and C6 sugars that can either be respired in glycolysis or enter the pentose phosphate pathway (PPP), complete for both oxidative as well as non-oxidative branches, for the production of nucleic acids or aromatic amino acids. Several putative TRAP-family dicarboxylic acid transporters were identified, suggesting C4 dicarboxylic acids could be used for energy production through enzymatic carboxylate degradation in the tricarboxylic acid (TCA) cycle, which is entirely present and highly conserved. C4 carbon compound degradation seems to

play a very important role in Msb3. Glycolate, glyoxylate and malate degradation pathways are present and complete. Dicarboxylic acids are likely among the preferred substrates for growth and may provide energy and a source of carbon for growth in planta.

Msb3 appears to be unable to utilize complex carbohydrate substrates, common in plant tissue. Although cellulose biosynthesis as well as glycogen biosynthesis (from ADP-D-glucose) pathways are present, degradation genes are not or the pathways are eroded, e.g. Msb3 possesses an endo-1,4-D-glucanase (bcsC; EC: 3.2.1.4) to catalyze the first step of cellulose degradation, hydrolysis of cellulose to cellodextrin; however, all other genes in the pathway are missing.

C1 oxidation and assimilation may play a role in this species as well: although only an incomplete formaldehyde assimilation pathway, as part of the serine pathway, was identified, oxidation pathways for formaldehyde, glutathione-dependent and thiol-dependent, are complete and even functionally redundant. Formate oxidation to CO_2 can eventually be achieved through some identified formate dehydrogenases. Additionally, an entire Calvin–Benson–Bassham cycle for CO_2-fixation in autotrophic organisms is present. However, genes are dispersed throughout the genome of strain Msb3 and all of the genes involved in this process are also involved in other processes, raising the question of its functionality.

Genes for the fermentation of pyruvate to acetate and to ethanol could also be identified. From the presence of the pathways outlined here we can infer a heterotrophic or potentially mixotrophic growth mode for this strain. Like P. xenovorans and P. phytofirmans the carbon utilization pathways in strain Msb3 are divers. All share central pathways for glycolysis/gluconeogenesis, TCA cycle, PPP and a wide variety of ABC transporters. Interestingly, however, only specific TRAP transporters were found among genes shared by strain Msb3 and P. phytofirmans PsJN but not P. xenovorans LB400, indicating that certain adaptations to a specific environmental niche may have reshaped even central carbon acquisition strategies.

Nitrogen Metabolism. The genome of strain Msb3 encodes an ammonium transporter (amtB) and a functional GS/GOGAT pathway: (glutamate synthase; glutamine synthetase). A glutamate dehydrogenase enables Msb3 to activate an ATP independent non-cyclic glutamate biosynthesis pathway, which is useful at high ammonia concentrations. In addition, reduction of nitrite to ammonia is possible through the action of a NADH-dependent nitrite reductase (nirBD) and assimilation through nitrate/nitrite transporters. An assimilatory nitrate reductase has not been found; therefore, reduction of nitrate to nitrite is not possible, indicating that nitrite can be used directly as a substrate for ammonia assimilation.

Like LB400, strain Msb3 encodes an entire nif nitrogen fixation gene cluster located on C2. This would enable the strain to convert atmospheric N_2 to ammonia through action of the multiprotein nitrogenase complex, comprised of an iron-molybdenum dinitrogenase (nifD and nifK), the dinitrogenase reductase subunit (nifH), ferredoxins, and maturation and regulatory factors. Although all nitrogenase enzymes are extremely sensitive to oxygen (Gallon, 1992), in vitro diazotrophy has unambiguously been shown in many plant-associated, non-legume-nodulating, rhizospheric and endophytic Paraburkholderia species, including P. unamae, P. tropica, P. silvatlantica, P. xenovorans, P. vietnamiensis and P. kururiensis (Martinez-Aguilar et al., 2008; Suarez-Moreno et al., 2012). Yet strategies to

limit oxygen exposure to the enzyme and general nitrogen fixation strategies/activities in planta, apart from those of legume-nodulating species, are unknown.

No nitrogen fixation genes have been found in PsJN, indicating that, among these closely related strains, there is strong selective pressure on this multi-protein gene cluster and conserving it may be necessary for the occupation of a specific niche.

Biosynthetic gene clusters. Other complete biosynthetic gene clusters include glycogen biosynthesis (glgABC), acetyl-CoA synthesis (aceEF), fatty acid (FA) activation and biosynthesis activation (accABC; fabDH) together with saturated FA elongation, as well as palmitate and palmitoleate biosynthesis (fabBGIVZ) and glutathione biogenesis (gshAB). Overall, strain Msb3 is a metabolically potent bacterium with many different strategies for survival.

Secondary metabolism. Secondary metabolism also seems to play an important role in the lifestyle of Msb3. We identified several genes involved in biosynthesis of a diverse set of secondary metabolites. On C2 we identified a non-ribosomal peptide synthetase (NRPS) consisting of a two-protein core cluster, a "amino acid adenylation enzyme/thioester reductase family protein" as well as a "non-ribosomal peptide synthase/amino acid adenylation enzyme" predicted by antiSMASH, and a border cluster containing an ABC-superfamily cyclic peptide transporter as well as all necessary transport system components including ABC-type Fe^{3+}-siderophore/hydroxamate permease and periplasmic components. Gene clusters predicted to produce some bacteriocins were detected on both chromosomes, including one responsible for linocin-M18 production.

Terpenoid biosynthesis is also prominently represented by three protein clusters, two of which encode a putative squalene synthase (SQS), responsible for the catalytic reduction of two farnesyl pyrophosphate molecules to squalene through the consumption of NADPH. Squalene is an acyclic triterpene with a tail-to-tail linkage between two 3-isoprene units and is a parent molecule of cyclic triterpenoids. The third gene cluster involved in terpenoid biosynthesis is a hpn (hopanoid biosynthesis) operon (hpnBCDEN, shc, nifB). It encodes enzymes for hopanoid biosynthesis, structural modifications and transport. Squalene-hopene cyclase (shc) can synthesize cyclic triterpenoids directly from squalene. Hopanoid lipids are thought to provide membrane stability and to mediate stress resistance under low pH and high osmotic pressure (Belin et al., 2018). Interestingly, many hopanoid-producing bacteria are capable of nitrogen fixation. While its supportive role in symbiotic nitrogen fixation has been unambiguously shown, it is also hypothesized to facilitate free living nitrogen fixation in Frankia spp. (Berry et al., 1993; Belin et al., 2018), which shield the reaction from oxygen through vesicles whose envelops consist of up to 90% bacteriohopanetetrol (BHT). Intriguingly, an analysis of hpnP and shc distributions in metagenomes also found that hopanoid production is enriched in plant-associated environments (Ricci et al., 2014). It is not only correlated with nitrogen fixation but also methanol utilization and seems to be a common trait among many non-nitrogen-fixing plant beneficial *Bacilli* (Racolta et al., 2012; Belin et al., 2018). The exact role of hopanoids in plant beneficial bacteria is not known but hypothesis include exchange of signals between bacteria and plants and the molecules being carriers for auxin-like compounds that affect plant development (their role in plant–bacteria interactions is comprehensively reviewed in (Belin et al., 2018)).

Finally, we identified a type I polyketide synthase (T1PKS) cluster on Plasmid 2. It is part of a larger gene cluster associated with capsule polysaccharide production and export. The synthesis of many complex polyketide antibiotics or immunosuppressants in bacteria is catalyzed by such multimodular PKSs. The core of each synthetic module consists of a ketosynthase (KS) an acyltransferase (AT) and an acyl carrier protein (ACP) domain. In downstream processes the resulting polyketide chain can be modified, resulting in a remarkable structural and functional diversity (Fischbach and Walsh, 2006). One such compound produced by *Burkholderia s.l.* species is rhizoxin. Producers are Mycetohabitans spp. and symbionts of the fungus Rhizopus sp. Without the symbiont, the fungus cannot make the polyketide that inhibits mitosis in rice plants (Partida-Martinez and Hertweck, 2005). Generally, these natural secondary metabolites play an important role in pathogenesis as well as symbiosis and their numerous possible applications in medicine and agriculture, including their use as biopesticides and anti-cancer drugs (Esmaeel et al., 2018), qualify them for further investigation.

Comparison of strain Msb3s genomic content to closely related strains

The isolation source of Msb3 hints at a plant-associated lifestyle. Although both metabolic versatility as well as the fact that it could easily be cultivated, suggest that a free-living life-style is possible and probable, consistent host colonization over space and time indicates that Msb3 is closely associated to its host. While the closely related strain PsJN displays similar behavior in regard to its host colonization (Sessitsch et al., 2005) as well as genome size, architecture and content (Mitter et al., 2013), the closest relatives of strain Msb3, LB400 and Ch1-1 only share the later. Both organisms have originally been isolated from soils and are known for the degradation of a large set of aromatic compounds (Vacca et al. (2005) for the isolation of Ch1-1; Hickey et al. (2012) for the genome and PAH degradation of Ch1-1; Goris et al. (2004) for the classification of LB400; Chain et al. (2006) for the Genome of LB400 and aromatic compound degradation). Some *P. phytofirmans* strains have also been isolated from soils and some *P. xenovorans* strains have been isolated from the plant rhizosphere (strains LMG 21720 and LMG 22943), indicating that there is a general tendency of this group of bacteria to interact with plants in some way. However, the close interaction observed for PsJN and Msb3 with their hosts, and their presence in the phyllosphere, are unique.

To dissect differences between these organisms, the whole gene complement of strain Msb3 was compared with that of PsJN and that of LB400, two strains for which detailed data on lifestyle, genome properties and metabolism exist.

Using the COGtriangle software all CDS from all three genomes were classified into clusters based on symmetrical best hits using an e-value of 10^{-9} as a threshold for BLAST hits and 70% for hit coverage. Numbers of CDS clustering together into a COG are shown in Supplementary Fig. 3. 4734 COGs are shared among all three strains, representing the core set of genes for this group of organisms, constituting their basic metabolism. As expected these clusters include genes for core metabolic and basic cellular processes as described above (see: Primary Metabolism). The close affiliation of LB400 and Msb3 is underlined by the 993 orthologs they share between themselves. Nevertheless, a high amount of CDS is not: LB400 shares 449 sequences with PsJN and has an additional 2084 CDS unique to its genome. Msb3, in turn, shares 302 CDS with PsJN and has 1440 unique genes. Unique genes of Msb3 include roughly 63% proteins of unknown function, making it difficult to derive

functional propositions. It should be noted, however, that 4.5% (65 CDS) of these carry the annotation ‘conserved exported protein of unknown function’, indicating that extracellular signaling and secretion could play a big role in the lifestyle of Msb3. Additionally, characterized proteins give insight into some unique niche adaptations: a fully functional TRAP-transporter system (OctPQM) for the import of C4-dicarboxylates, various ABC-type transporters, putatively transporting di- and oligopeptides as well as an excessive phosphate transport system (e.g. pstABCS) and proline/ glycine-betain transport system were found within the strain-specific-genome of Msb3. Furthermore, genes for many NodT-like efflux transporters and some type VI seceretion system (T6SS) secreted proteins were identified, underlining the importance of secretion and signaling.

To identify genes possibly involved specifically in a plant associated lifestyle we investigated clusters shared between Msb3 and PsJN, but absent in LB400. Again, 302 common elements include many conserved exported proteins of unknown function but, most notably, a fully functional T6SS. Other shared COGs contain complete teichuronic acid biosynthesis and export gene clusters (TuaH; TagGH) as well as flagellar motor proteins and a whole cassette of pilus assembly proteins and secretins (CpaCBAFE) that belong to the Flp/Tad pilus system. Evidence for the involvement of tad loci in colonization and pathogenesis has been presented multiple times since the locus was described (reviewed in Tomich et al. (2007)) and could also play a role in an endophytic lifestyle of Msb3. It should be noted however, that also LB400 carries a complete Tad system in its genome (rcpA, TadABCVZ) that is shared with both, Msb3 and PsJN. Tad loci are present in many Burkholderia s.l. species and may contribute to their general role as host associated organisms. In LB400 it may mediate its ability to adhere to the plant root and thus contributes to its lifestyle as a PGPR.

Although LB400 presumably has a versatile environmental niche (soil and plant rhizosphere) it is most famous for the degradation of PCB and many other aromatic, chlorinated compounds. The natural ability to degrade aromatic compounds could have originated from degradation of root exudates and root turnover. P. xenovorans strain LB400 is enriched in aromatic catabolic pathways, which are located on genomic islands. The genes provide the strain with biphenyl, 2-aminophenol, 3-chlorocatechol, and other catabolic capacities (Chain et al., 2006). Msb3 shares the bphA1 and bphA2 orthologs ‘ring hydroxylating enzyme’ alpha SU and beta SU with LB400, but not with other representatives of the group. They are contained in a genetic island on Chr2 and the alpha SU displays 79.83% identity to the ‘aromatic ring-hydroxylating dioxygenase subunit alpha’ (RefSeqID: WP_011493740.1) from LB400. Both are orthologs of the bphA1 (Biphenyl 2,3-dioxygenase subunit alpha) gene of Rhodococcus jostii (strain RHA1) (UniprotID: Q53122) with an identity of 30.74% and 31.2% for Msb3 and LB400, respectively. BphA plays a major role in aromatic hydrocarbon catabolism using biphenyl or chlorine-substituted biphenyls as substrates. The presence of both, SU alpha and SU beta, shows that Msb3 has some potential for the degradation of complex aromatic compounds, although the diversity of these pathways and the metabolic versatility is considerably lower than in LB400. Strain Ch1-1 also carries an ortholog of bphA1, however, it is only 31.16% identical to the one of Msb3. Accessory genes for poly-aromatic hydrocarbon (PAH) catabolism are largely missing from the Msb3 genome or the pathways are eroded, indicating that it may not be part of its primary lifestyle.

Judging from the isolation source of strain Msb3 together with the tendency to conserve genes involved in host association and the lack of diverse PAH degradation pathways, unlike its closest relatives LB400 and Ch1-1, it seems likely that the strain is indeed, for the better

part of its life, associated to a plant host, the presence of core genes in the degradation of aromatic compounds could be related to degradation of complex, plant derived compounds.

Supplementary References

Belin, B.J., Busset, N., Giraud, E., Molinaro, A., Silipo, A., and Newman, D.K. (2018). Hopanoid lipids: from membranes to plant–bacteria interactions. *Nature Reviews Microbiology* 16**,** 304. doi: 10.1038/nrmicro.2017.173.

Berry, A.M., Harriott, O.T., Moreau, R.A., Osman, S.F., Benson, D.R., and Jones, A.D. (1993). Hopanoid lipids compose the Frankia vesicle envelope, presumptive barrier of oxygen diffusion to nitrogenase. *Proceedings of the National Academy of Sciences* 90(13)**,** 6091-6094. doi: 10.1073/pnas.90.13.6091.

Callahan, B.J., McMurdie, P.J., Rosen, M.J., Han, A.W., Johnson, A.J.A., and Holmes, S.P. (2016). DADA2: High-resolution sample inference from Illumina amplicon data. *Nature methods* 13(7)**,** 581-583. doi: 10.1038/nmeth.3869.

Caporaso, J.G., Kuczynski, J., Stombaugh, J., Bittinger, K., Bushman, F.D., Costello, E.K., et al. (2010). QIIME allows analysis of high-throughput community sequencing data. *Nature methods* 7(5)**,** 335-336. doi: 10.1038/nmeth.f.303.

Chain, P.S., Denef, V.J., Konstantinidis, K.T., Vergez, L.M., Agullo, L., Reyes, V.L., et al. (2006). Burkholderia xenovorans LB400 harbors a multi-replicon, 9.73-Mbp genome shaped for versatility. *Proc Natl Acad Sci U S A* 103(42)**,** 15280-15287. doi: 10.1073/pnas.0606924103.

Esmaeel, Q., Pupin, M., Jacques, P., and Leclère, V. (2018). Nonribosomal peptides and polyketides of Burkholderia: new compounds potentially implicated in biocontrol and pharmaceuticals. *Environmental Science and Pollution Research* 25(30)**,** 29794-29807. doi: 10.1007/s11356-017-9166-3.

Fischbach, M.A., and Walsh, C.T. (2006). Assembly-Line Enzymology for Polyketide and Nonribosomal Peptide Antibiotics: Logic, Machinery, and Mechanisms. *Chemical Reviews* 106(8)**,** 3468-3496. doi: 10.1021/cr0503097.

Gallon, J.R. (1992). Reconciling the incompatible: N2 fixation And O2. *New Phytologist* 122(4)**,** 571-609. doi: 10.1111/j.1469-8137.1992.tb00087.x.

Goris, J., De Vos, P., Caballero-Mellado, J., Park, J., Falsen, E., Quensen, J.F., 3rd, et al. (2004). Classification of the biphenyl- and polychlorinated biphenyl-degrading strain LB400T and relatives as Burkholderia xenovorans sp. nov. *Int J Syst Evol Microbiol* 54(Pt 5)**,** 1677-1681. doi: 10.1099/ijs.0.63101-0.

Hickey, W.J., Chen, S., and Zhao, J. (2012). The phn Island: A New Genomic Island Encoding Catabolism of Polynuclear Aromatic Hydrocarbons. *Front Microbiol* 3**,** 125. doi: 10.3389/fmicb.2012.00125.

Martinez-Aguilar, L., Diaz, R., Pena-Cabriales, J.J., Estrada-de Los Santos, P., Dunn, M.F., and Caballero-Mellado, J. (2008). Multichromosomal genome structure and confirmation of diazotrophy in novel plant-associated Burkholderia species. *Appl Environ Microbiol* 74(14)**,** 4574-4579. doi: 10.1128/AEM.00201-08.

Mitter, B., Petric, A., Shin, M.W., Chain, P.S., Hauberg-Lotte, L., Reinhold-Hurek, B., et al. (2013). Comparative genome analysis of Burkholderia phytofirmans PsJN reveals a wide spectrum of endophytic lifestyles based on interaction strategies with host plants. *Front Plant Sci* 4**,** 120. doi: 10.3389/fpls.2013.00120.

Partida-Martinez, L.P., and Hertweck, C. (2005). Pathogenic fungus harbours endosymbiotic bacteria for toxin production. *Nature* 437(7060)**,** 884-888. doi: 10.1038/nature03997.

Racolta, S., Juhl, P.B., Sirim, D., and Pleiss, J. (2012). The triterpene cyclase protein family: A systematic analysis. *Proteins: Structure, Function, and Bioinformatics* 80(8)**,** 2009-2019. doi: 10.1002/prot.24089.

Ricci, J.N., Coleman, M.L., Welander, P.V., Sessions, A.L., Summons, R.E., Spear, J.R., et al. (2014). Diverse capacity for 2-methylhopanoid production correlates with a specific ecological niche. *Isme j* 8(3)**,** 675-684. doi: 10.1038/ismej.2013.191.
Sessitsch, A., Coenye, T., Sturz, A.V., Vandamme, P., Barka, E.A., Salles, J.F., et al. (2005). Burkholderia phytofirmans sp. nov., a novel plant-associated bacterium with plant-beneficial properties. *Int J Syst Evol Microbiol* 55(Pt 3)**,** 1187-1192. doi: 10.1099/ijs.0.63149-0.
Suarez-Moreno, Z.R., Caballero-Mellado, J., Coutinho, B.G., Mendonca-Previato, L., James, E.K., and Venturi, V. (2012). Common features of environmental and potentially beneficial plant-associated Burkholderia. *Microb Ecol* 63(2)**,** 249-266. doi: 10.1007/s00248-011-9929-1.
Tomich, M., Planet, P.J., and Figurski, D.H. (2007). The tad locus: postcards from the widespread colonization island. *Nat Rev Microbiol* 5(5)**,** 363-375. doi: 10.1038/nrmicro1636.
Vacca, D.J., Bleam, W.F., and Hickey, W.J. (2005). Isolation of soil bacteria adapted to degrade humic acid-sorbed phenanthrene. *Appl Environ Microbiol* 71(7)**,** 3797-3805. doi: 10.1128/AEM.71.7.3797-3805.2005.

Supplementary Figures

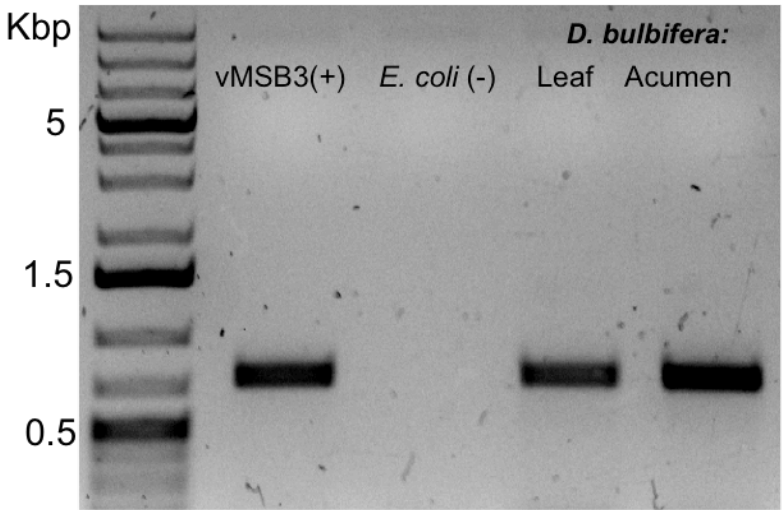

Supplementary Figure 1 | Amplification of gyrB as a marker gene for *Burkholderia* s.l. in *D. bulbifera* DNA extracts. DNA extracted from a pure culture of strain Msb3 (vMSB3) was used as a positive control and *Escherichia coli* dh5 alpha DNA (*E. coli*) was used as a negative control. DNA extracted from the leaf lamina and from the leaf acumen of *D. bulbifera* was included in the analysis. Members of *Burkholderia s.l.* were detected in both fractions.

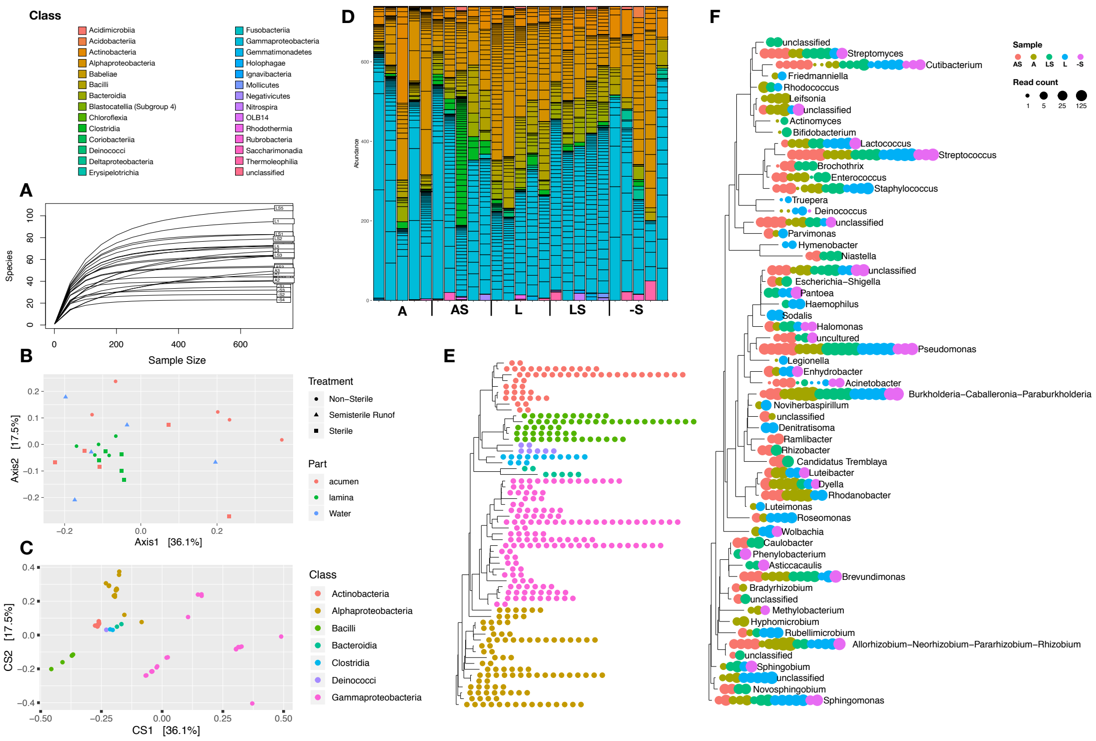

Supplementary Figure 2 | D. bulbifera leave amplicon sequencing. A, rarefaction curves of rarefied data after removal of plant derived sequences. B, principal coordinate analysis (PCoA) that was performed on the normalized ASV tables using pairwise, normalized, weighted UniFrac distances between all samples and the first two principal coordinates were visualized. C, most influential genera responsible for the separation along axis 1 and 2 in B, each dot represents one genus which are colored by class. D, relative distribution of bacterial classes within individual samples. Sample type is indicated on the x-axis, the color code for D is positioned on the top left corner. Shown is the dataset that was rarefied to 729 reads per sample before ASVs prevalent in less than 5 % of all samples were removed. In E the class distribution can be observed within a phylogenetic tree after removal of ASVs with low prevalence: each tip represents a bacterial genus whose occurrence in one sample is indicated by one circle. The fill color of the circle corresponds to the bacterial class using the same color code as C. F displays the same tree and the tips are labeled with the respective genus. Prevalence and abundance of the genus are indicated by the circles next to the tip label: a dot represents a sample and its color corresponds to sample type. The size of each circle corresponds to the abundance in that sample in read counts within the rarefied dataset.

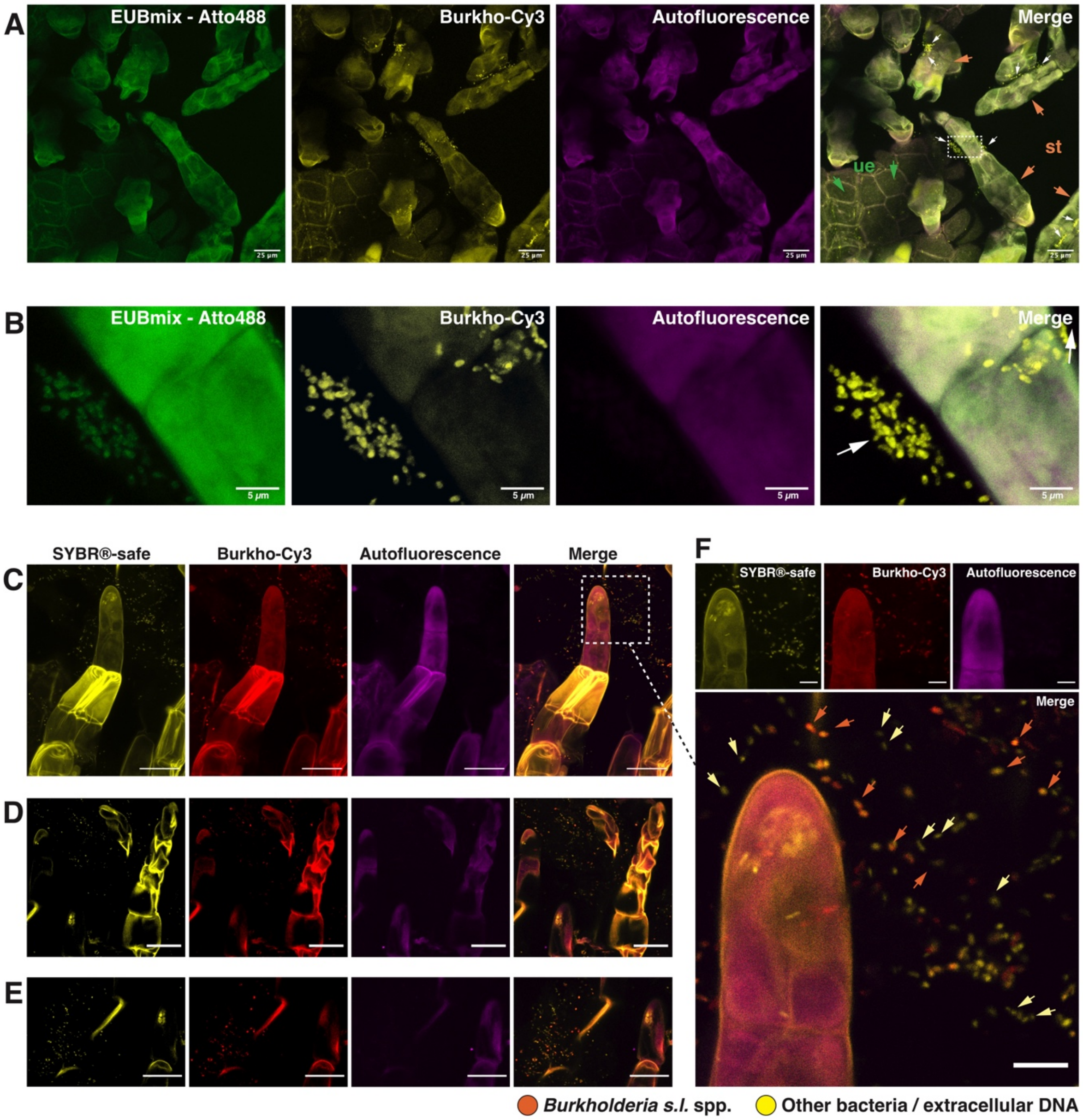

Supplementary Figure 4 | Visualization of bacteria and *Burkholderia s.l.* spp. on the surface of leaf acumens of *Dioscorea bulbifera* (potato yam) by DOPE-FISH/CLSM microscopy. **A** & **B** display original images corresponding to those shown in **Fig. 3**, before channel dye separation. EUBmix and Burkho probes were applied onto leaf acumens sequentially and the upper surface of the acumen, which is densely covered with secretory trichomes, was visualized (**A**). *Burkholderia s.l.* spp. (white arrows), labelled with both EUBmix (green channel) and Burkho probes (yellow channel), can be observed clustering on the sides of, or in between, secretory trichomes (**st**; orange arrows) emerging from the upper epidermis (**ue**; green arrows) of the leaf acumen. In **B** a high-resolution image of a cluster on the side of a trichome can be seen, all bacterial cells shown are labelled with the *Burkholderia s.l.* specific probe.

Because of the weak fluorescence intensity of the EUB-probe mix, compared to the plant background, we decided to combine a SYBR®-safe general DNA staining approach with DOPE-FISH using the Cy3 labelled Burkho probe to detect and separate other microbiota (or extracellular DNA) from Cy3 labelled cells (**C-F**). **C-E** each show a region, in which we managed to detect signals originating from microbes (or DNA particles) other than

Burkholderia s.l. species. While the later are still quite abundant within all 'microbial hotspots', other cell like structures can be observed as well, emitting only the SYBR®-safe associated signal, displayed in yellow. A magnification view of **C** is presented in **F**. Cy3 and SYBR®-safe labelled cells are indicated by orange arrows, other putative bacteria by yellow arrows. Scale bars: 20 µm in **C-E**; 5 µm in **F.**

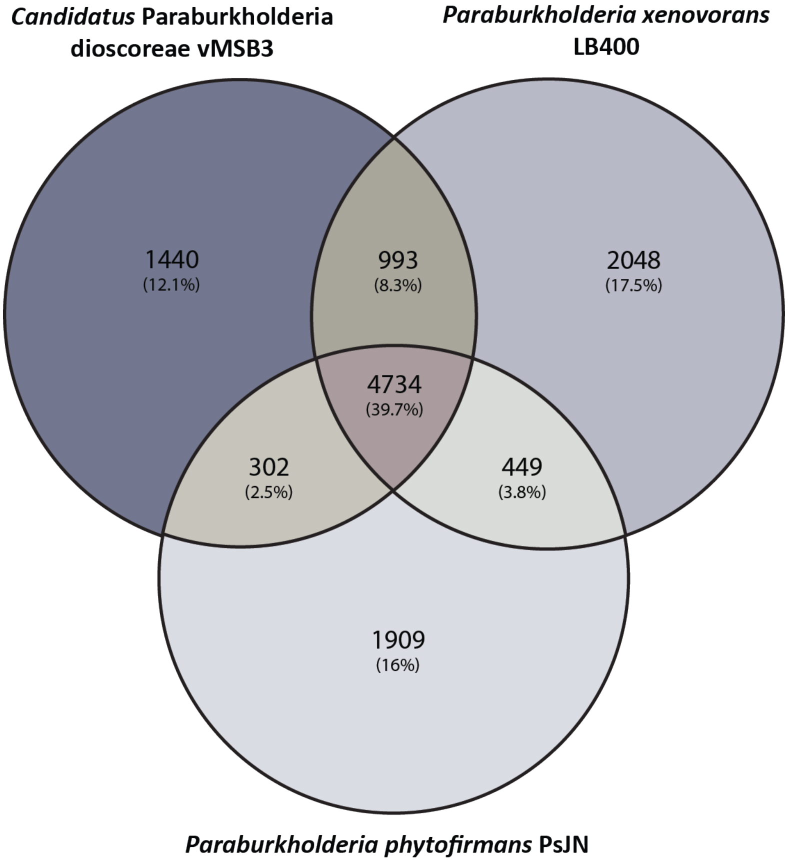

Supplementary Figure 4 | Core- vs. Pan-Genome of closely related *Paraburkholderia* species.

Clusters of orthologous groups of proteins (COGs) have been built using COGtriangles. Symmetrical best hits (e=10^{-9}; c=0.7) between the genomes included in the analysis were assigned to the same unique COG. Shown is the number of COGs shared by all genomes included in the analysis (center), constituting the core genome of the group. Also visible are the numbers of COGs unique to each genome (non-overlapping parts of the circles) and the number of COGs shared only between two out of the three genomes included (overlaps of two circles). Together this represents the pan genome of the three organisms whose genomes were included: *Paraburkholderia* sp. Msb3, *P. xenovorans* LB400 and *P. phytofirmans* PsJN
